# Revisiting health promotion settings: An innovative model from Sri Lanka to integrate healthy settings using mHealth

**DOI:** 10.34172/hpp.2022.04

**Published:** 2022-05-29

**Authors:** Millawage Supun Dilara Wijesinghe, WM Prasad Chathuranga Weerasinghe, Balangoda Muhamdiramlage Indika Gunawardana, RM Nayani Umesha Rajapaksha, VCN Vithana, SASC Karunaratne, Dinesh Koggalage, Palitha Karunapema

**Affiliations:** ^1^Consultant Community Physician, Health Promotion Bureau, Colombo, Sri Lanka; ^2^Registrar, Health Promotion Bureau, Colombo, Sri Lanka; ^3^Senior Registrar, Health Promotion Bureau, Colombo, Sri Lanka; ^4^Medical Officer, Health Promotion Bureau, Colombo, Sri Lanka; ^5^Deputy Director, Health Promotion Bureau, Colombo, Sri Lanka; ^6^Director, Health Promotion Bureau, Colombo, Sri Lanka

**Keywords:** Health promotion, Mobile applications, Telemedicine

## Abstract

The health promotion settings approach has been recognised as an effective method of health promotion in the recent era, and mobile health (mHealth) is a highly evolving field in the health sector. The health promotion settings are shifting the focus away from the individuals and moving towards a more holistic model of health promotion. We identified five settings in Sri Lanka to promote a mHealth model, including villages, schools, preschools, workplaces, and hospitals. The specified model using mHealth helps monitor the activities at various levels of healthcare, including regional, district and national levels. The model also maps the location of the healthy settings, which provide a visual picture to the policymakers, helpful in planning and decision-making.

## Introduction

Health Promotion is defined as the process of enabling people to increase control over and to improve their health.^1^ According to the Ottawa Charter, the core action areas of health promotion include ‘building healthy public policy, creating supportive environments, strengthening community action, developing personal skills, and reorienting health services. Supportive environments are recognised as one of the significant health promotion action areas strengthening many other core action areas in the charter by changing patterns of life, work and leisure that have a significant impact on health. Furthermore, developing ‘healthy settings’ as supportive environments to strengthen the community action enhances the empowerment of the communities, their ownership and control over their health.^1^ The settings approach provides a powerful methodological tool for promoting health within the context where health promotion occurs. It is, in essence, where the people live, work, and establish networks with others. Settings can differ from poorly defined communities to distinct communities, organisations, schools, and hospitals to workplaces.^2^

Health promotion initiatives are becoming increasingly complex from planning to implementation and comprise a mix of strategies aiming to achieve individual behaviour changes and much broader environmental changes.^3^ Therefore, the settings approach is essential in addressing inequalities and inclusion, place-shaping, and systems-based responses to complex health problems.^4^ Furthermore, Whitelaw et al proposed five types of setting-based health promotion concepts,^5^ starting from a ‘passive model’ to a ‘comprehensive/structural’ model. The core perspectives of the ‘passive model’ include a notion that the problem and solution rest within the behaviour and actions of individuals to a more ‘comprehensive/structural’ model, where the solution lies in the setting. Although the usefulness of the settings approach is well documented in health promotion, it is also noted that there are inherent challenges in evaluating the approach’s effectiveness in using known methodologies. These challenges can be explicitly stated how healthy settings relates to the known evidence for health promotion, its diversity of conceptual understandings and real-world practice, and the difficulty of evaluating ecological whole-system methods.^6^ Upon further applying the above challenges to country-specific context, there is limited evidence base in Sri Lanka regarding healthy settings because of problems such as more funding being allocated for issue-based (i.e. disease-specific) than settings-based initiatives.

Furthermore, diversity of conceptual understanding and real-life practice of the concept of the settings at national, regional and grassroot levels is still problematic. Most importantly, disagreements in mechanisms of evaluation due to the different types of the settings, size of the settings, and whether the setting is formal (such as institutions) or informal (such as villages or communities) is also one of the key challenges the health system is currently facing. The present situation is further worsened by the existing inefficient paper-based systems used for monitoring health promotion initiatives.

Digitalising health is recognised as a valuable opportunity to achieve Sustainable Development Goals. Furthermore, digitalising health is identified as a key step in the pathway of accelerating to the “Triple Billion Target”,^7^ of the World Health Organization, that is planned to be achieved by 2023.^8^ Digital health is a broad concept divided into several sub-sections such as health information technology, mobile health (mHealth), telemedicine and telehealth, wearables, and personalised medicine.^9^ With the rapid development and access to information and communication technology, the formulation of long-term strategic planning and policy development for e-health is identified as a priority for every country to ensure affordable, universal, and equitable access to health by the World Health Assembly 2005.^10^

Sri Lanka has a high potential for the successful implementation of mHealth start-ups with the recent advancement of information and communication technology and its access. According to the latest statistics, 30.41 million mobile connections are activated in Sri Lanka. It is 141.7% compared to the population, showing that many Sri Lankans own more than one mobile connection. There was a 2.1% growth in connections last year, and 79.8% are broadband connections (3G-4G). Among smartphone users, the vast majority (91.53%) use the Android operating system, and 8.19% are IOS users.^11^ Current local internet penetration is 50.8% of the population, and there is a 7.9% growth compared to January 2020. Mobile phones are the most widely used device (64.0%) to connect to the internet in the country, with desktop and laptop computers coming in second (34.7%).^11^ Although there are no estimates of mobile application usage in Sri Lanka, mHealth applications have been identified as a vehicle for health system strengthening in the country.^12,13^ Therefore, mHealth is a promising tool where health interventions can be successfully monitored.

Although the ‘healthy settings’ idea is not new to the Sri Lankan health promotion landscape, the concept was not formally recognised and documented until recently. The first documented evidence of the importance of health promotion settings was found in the newly developed National Health Promotion Policy in 2010,^14^ and the concept was revitalised lately with the Health Promotion Strategic Plan 2020-2025. The Health Promotion Bureau (HPB) of Sri Lanka is currently establishing, strengthening, and popularising the concept of healthy settings in the country. Though many activities were conducted regarding health promotion (HP) settings, monitoring the activities and evaluating the impact of the conducted activities are not occurring at the national or district level. Therefore, as a monitoring tool, the use of mHealth was proposed to achieve it.

## The development process of the mHealth model

**Figure 1:**
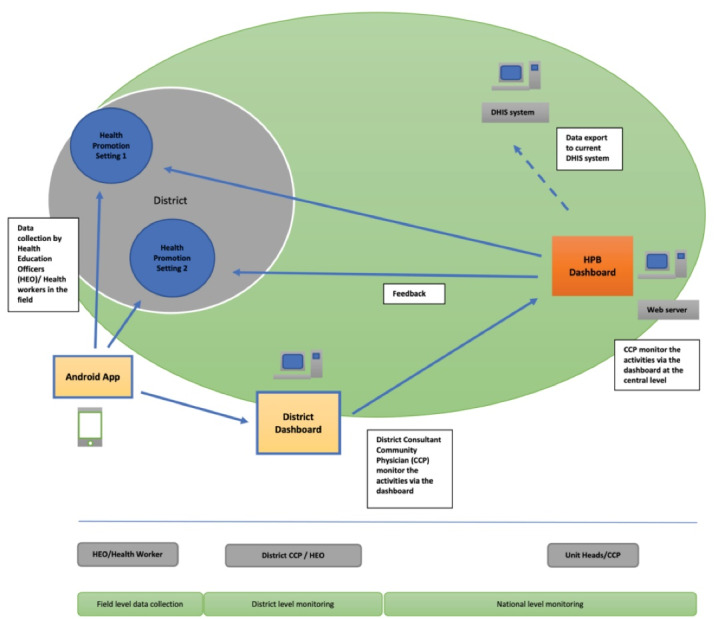
The framework for health promotion settings model.

There were four stages in the development process, including ‘identification of suitable health promotion settings’, ‘development of the Android app & dashboard’, ‘training staff’, and ‘monitoring of activities’. The overall framework of the model is given in Figure 1.

## Identification of suitable health promotion settings

A health promotion setting can vary from place to organisation to community.^2^ However, for this model, the HPB identified existing five settings after a detailed analysis of attributes, including available persons, thinking patterns and mode of operation, implicit social norms, hierarchies of power, accountability mechanisms, local moral, political and organisational culture, and physical and psychosocial environment.^15^ Therefore, preschools, schools, workplaces, hospitals, and villages were identified as the suitable places to implement the approach based on the above criteria. Before developing the model, the development team conducted four focus group discussions (FGDs) with the key stakeholders (National and Regional Consultant Community Physicians, Medical Officers of Health, District Health Education Officers, Public Health Inspectors, Public Health Midwives, Educational authorities, Community-based organisations) regarding the need.

## Development of the Android app & dashboard

We employed an android app development company to develop the mobile app based on the technical requirements given by the HPB. Eight FGDs were held to finalise the data input, app menu and app user interface. The android application was developed and shared with the HPB before deployment. Following the development, the usability testing of the app was evaluated by the HPB, and a pilot test was conducted before the implementation. The developed app is available in the Google Play android app store as a free download.^16^

A web-based monitoring dashboardwas developed to monitor and moderate user activities and generate reports parallel to the mobile application.^17^ The system was pretested before giving access to the supervising officers and upgraded two times. In addition, the developer provides continuous support to fix minor bugs as soon as detected by a dashboard user.

The app provides the following functionality. First, it registers the location and the details of the healthy settings (with the user details). Regular events can be added daily to the app once the regional or central level approves the setting. Each updated event is shared with the total user base of the app. In addition, regional and central level administrators can create news items and share them with app users. Second, the app offers general productivity improvement and information retrieval functionality in the setting. It also acts as a success sharing portal. The users can learn from the success stories of others and replicate them in their settings. The app also helps users keep up to date with the latest information on health promotion activities. Finally, the whole system act as a visual tool (dashboard) for policymakers to keep track of the progress of each setting. It also provides details about how active each setting in a district is at a given time.

## Training staff

The app development company trained all district-level staff who are involved in establishing health promotion settings. During the training, participants were asked to download the app by searching the ‘Health Promotion App’ by screening the QR code with link.^16^ Regular refresher training has been conducted for health staff for two years. The HPB switched the training to virtual-based sessions during the COVID-19 pandemic. Health Education Officers (HEOs) working at the district level were trained as the master trainers to implement a cascade training mechanism. Apart from these regional training sessions, regular national level virtual training sessions were conducted by the HPB technical team. The HPB prepared two training guides for the mobile app, and we shared the dashboard with the master trainers (Supplementary files 1 and 2).

## Monitoring of activities

A district health education officer identified a key person from each setting in the districts to register and enter data into the mobile application. The added new settings need to be validated from the district level and entered into the system by the district HEO. The validated data was cross-checked at the national level. Figure 1 illustrates the workflow of the app. A designated Consultant Community Physician at the HPB, Ministry of Health, monitored the activities occurring in the settings. Higher-level users used a web-based dashboard to monitor the activities of the mobile application with several levels of access (Regional/District/National). In addition, the locations and the activities of the established settings in the country are being monitored by the HPB.

## Initial uptake

After the training, the app was downloaded by 1000+ users. There are currently 556 active users (to date) who are regularly updating the activities. The highest uptake was seen with village-level settings, followed by hospitals and preschools. There was a limitation of activities because of the COVID-19 pandemic with lockdowns in the country for the past year, with schools, preschools and workplaces being closed most of the time. Therefore, the active promotion of the app was ceased until the COVID-19 control measures were lifted. The new events and the news features were the most popular among app users.

## Role of mHealth in strengthening healthy settings

Health promotion through a healthy setting is a well-known, widely used, and top-rated approach. The primary aim of the healthy setting is to create a supportive environment to promote and improve health, using fundamental principles of flexibility, community participation, partnership, empowerment, and equity. Thus, the healthy setting can be used as a successful approach to promote health. It can incorporate the holistic and multidisciplinary method to integrate the actions across risk factors.^18^ A wide range of mHealth technologies, from a simple reminder for patients to complex monitoring and evaluation tools or data collection applications, is expanding rapidly in lower-income countries.^19^ Health education/awareness, supporting diagnosis inpatient care, facilitation of health-related data collection, technologies related to remote patient monitoring, telemedicine, improving patient compliance, and supporting chronic disease management are major aspects of mHealth solutions.^20^ Using mHealth technology in monitoring health promotion activities (such as healthy settings) provides a cost-effective way of improving efficiency and effectiveness. However, how widely mHealth interventions are used depends on the smartphone penetration of a country.

Currently, 48.2% of the global population uses smartphones,^21^ and the majority (72.73%) is running with the Android operating system.^22^ Mobile applications can be classified under various subgroups: personal health, mental health, telehealth, neurotech, sleep tech, fem tech, diet and nutrition, diet and fitness, fitness and sports, diagnostics and prognostics, and COVID-19.^23^ People have started to use m-health apps 40% more frequently with the COVID-19 pandemic, probably because of the risk of physical encounters with healthcare professionals and disruption of the healthcare services.^24^ Apart from user engagement, most modern apps can map the geolocation of the users, which helps measure coverage of any health intervention. Geographic information systems (GISs) are identified as a powerful tool to visualise data by spatial referencing, visually disseminate information, as a training tool, and link the activities with measurable public health benefits in communities and districts.^25^ In addition, GIS can be considered an ‘enabling technology’ that can answer pertinent questions related to evaluating community-based health promotion interventions (such as healthy settings) conducted at the field level. For example, a visual map can be produced using GIS with community profiling (with identified health issues) in the districts, which helps plan and monitor interventions more efficiently.^26^ However, the success of any mHealth intervention depends on its adaption in the community.

## Success factors of the mHealth model

Adaptation of a mHealth intervention is governed by the theories of perceived usefulness and perceived ease of use.^27^ We developed the app to be valuable to the users to showcase their healthy settings and their events to other users in the community. The app’s application interface was satisfactory because it was developed and displayed based on the community feedback. Culturally embedded factors were explored to support the community’s adaptation and acceptance.^28^ Moreover, as we have already developed a supportive environment for healthy settings, it is more convenient for us to introduce this mobile app and get their cooperation to use it in parallel with their routine work. Furthermore, since there was already an existing robust public health structure, it was easy for us to implement the mobile app.

In addition, we have conducted several hands-on training sessions for the public health staff (who are directly in contact with the health promotion settings) before introducing this app for the members of the health promotion settings. The HPB prepared two user manuals for easy reference for the technical matters encountered during instalment and use of the HP app and the web dashboard. These manuals increased the perceived ease of use among the app users.^27^

Furthermore, we built a proper communication channel with users who wish to install the HP app and the HPB via telephones or emails. The maintenance team at the HPB is ready to answer the issues anytime. The app development team handled the other technical problems that were beyond the maintenance team at the HPB. Even with a minimal level of IT knowledge, any member in the HP settings can use this app because of its user-friendly nature.

## Evaluation of mHealth model and key challenges

Digital health interventions are useful to generate evidence for health promotion.^29^ Nevertheless, it is important to evaluate whether it achieves its intended goals. We have evaluated the mHealth model we developed, which measures implementation fidelity towards attaining its goals using the following quality standards^30^; does the system meet the defined technical specifications, is the system stable and error-free, does the system perform its intended tasks consistently and dependably, are there variations in implementation across or within sites, and benchmarks for deployment are being met as expected.

With regards to the developed system meeting defined technical specifications, although implementing the mobile app met the stipulated expectations, the new information system performance is not straightforward because of the many influencing factors. Development of a mobile app to suit whatever the operating system is challenging. At present, the developed HP app is supported only by the android operating system. In low resource settings, maintenance and further improvement of the app is challenging because of finical constraints. With limited resources, further improvement of the supportive environment with relevant facilities, such as uploading videos, documents, and one-to-one chats or messages, is challenging. In addition, the sustainability of the app needs to be strengthened with timely improvement and regular feedback from the users.

Moreover, regarding the system performing its intended tasks consistently and dependably, the speed of the internet connections and the signal strengths vary in different geographical locations. Thus, problems can arise with the users. They may be resistant to using new apps in such backgrounds. As a solution to these problems, there is room for the development of an offline version. However, it is a challenging task with limiting resources.

Furthermore, people who refuse smartphones and new technologies and wish to continue the traditional paperwork need to be addressed differently. In addition, it is challenging to integrate the other health apps with the health promotion app due to a limited number of healthcare workers. Moreover, healthcare workers’ priorities are different, depending on the fields and the currently involved programmes.

Although the system was performing well in the aspects of stability and met all the required benchmarks, there were variations in implementation across or within sites. This variation was primarily because of the COVID-19 pandemic restricting the movement due to lockdowns.

## Conclusion

We highly recommend using the HP app in Sri Lanka to improve the health promotion settings because of its user-friendly nature, the ability to deliver related information fast, and easy monitoring of activities related to health promotion. It provides a clear visual picture to the policymakers that are helpful in planning and decision-making. Furthermore, it gives the much-needed engagement with the communities via interactive two-way communication. It will be an essential initial step to replace the old-fashioned paper works of community health workers.

## Acknowledgments

The authors would like to acknowledge the UNFPA for their support in developing the health promotion app and the dashboard.

## Authors’ contributions

MSDW was responsible for conceptualization and writing of the manuscript. WMPCW, BMIG, RMNUR, VCNV, SASCK, DK and PK reviewed the draft manuscript. All authors reviewed and approved the final manuscript.

## Funding

No funding was received from any institution or department.

## Ethical approval

Not applicable.

## Competing interests

None declared.

## Supplementary Materials

Supplementary files 1 and 2 contain training guides for the mobile app and the dashboard.
