# Supplementary material for: Revisiting health promotion settings: An innovative model from Sri Lanka to integrate healthy settings using mHealth
Source: Health Promot Perspect. 2022 May 29;12(1):28–33. doi: 10.34172/hpp.2022.04 (PMC9277291; doi:10.34172/hpp.2022.04)
Supplement: Supplementary file 1 [file hpp-12-28-s001.pdf]

Instruction Manual for Users of the Health Promotion App

Ver 1.1

Family Health & Nutrition Communication Unit

Health Promotion Bureau

Table of Contents

A. How to download and install the Health Promotion App 3
B. How to open the app 5
C. How to register yourself as a User 6
D. How to register a Mothers' Support Group 8
E. How to add an event conducted by the Mothers' Support Group 11
F. How to look at the latest events 13

A: How to download and install the Health Promotion App

Method 1

1. Go to **Google Play Store**
2. Type '**Health Promotion App**' on the search bar

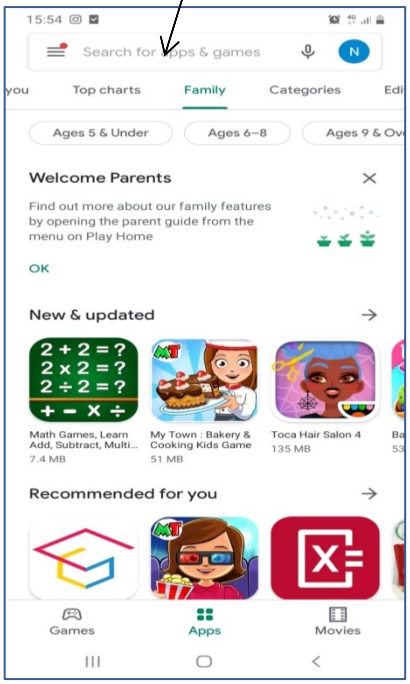

3. Select the following icon

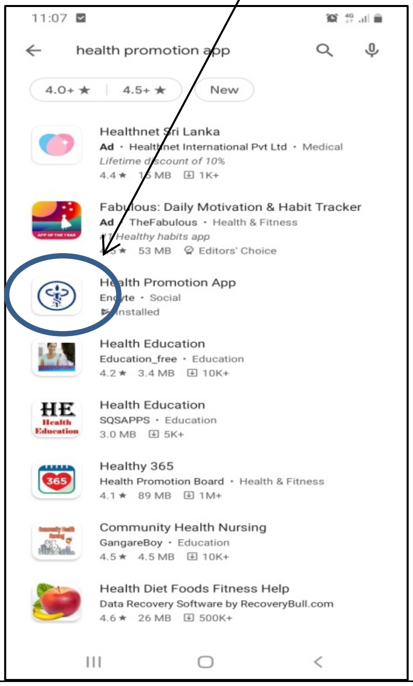

Method 2

1. Scan the given QR code

2. Click on the URL
3. You will be taken to the screen below

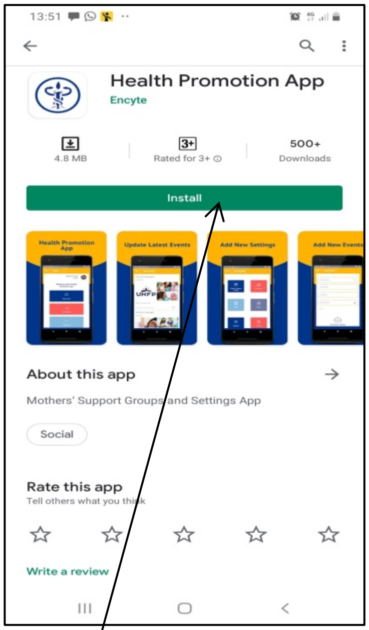

4. '**Install'** the app.

5. Once installation is complete, the icon will appear in the home screen of the smart phone similar to below

4. You will be taken to the screen below

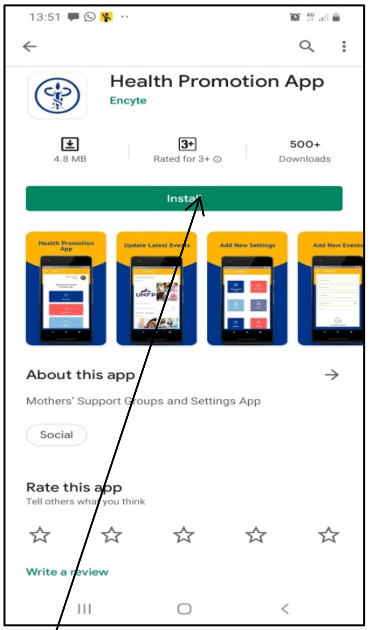

5. '**Install**' the app.
6. Once installation is complete, the icon will appear in the home screen of the smart phone similar to below

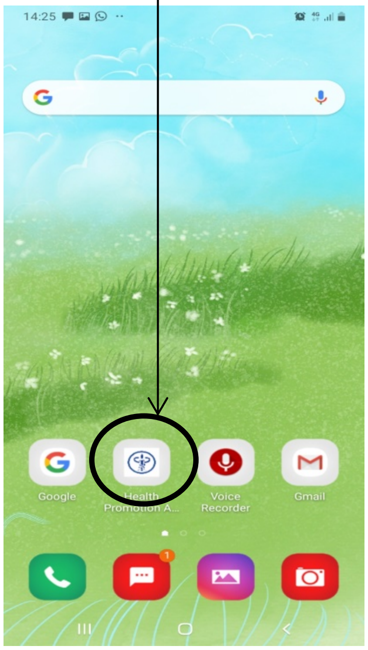

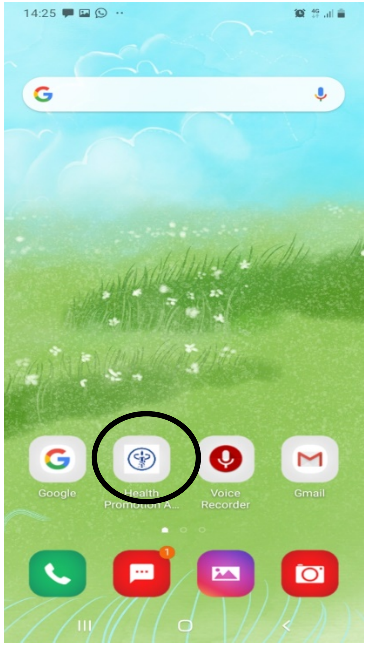

B: How to open the app

1. Click on the **Health Promotion App** in the home screen.

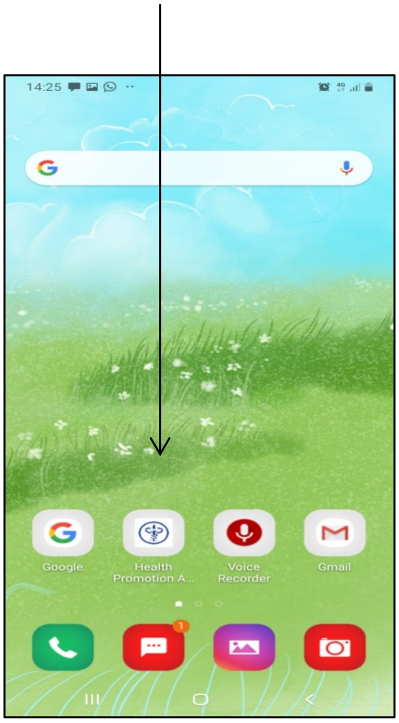

2. The following screen will appear

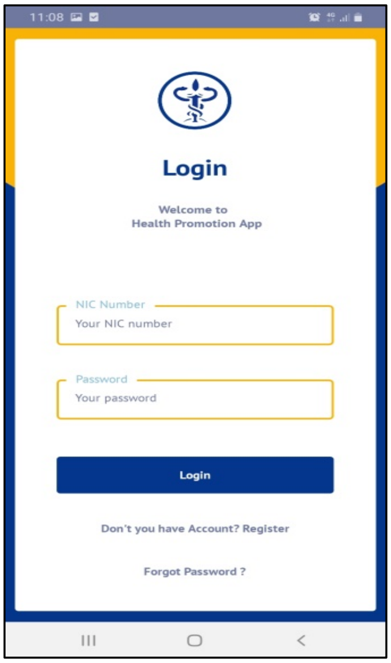

C: How to register yourself as a User

1. Open the Health Promotion App
2. Click on **Register** to register yourself as a User

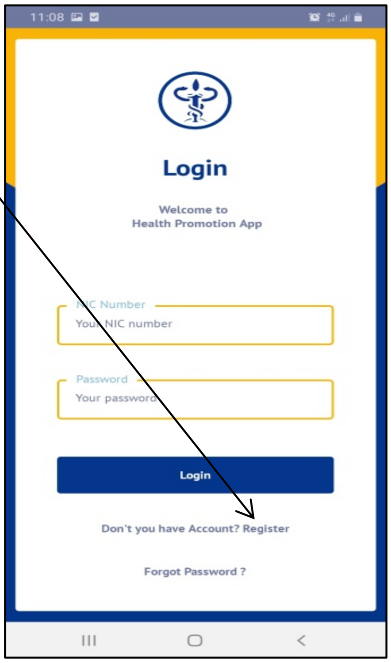

3. Confirm your location in the given screen and select ' **Next**'

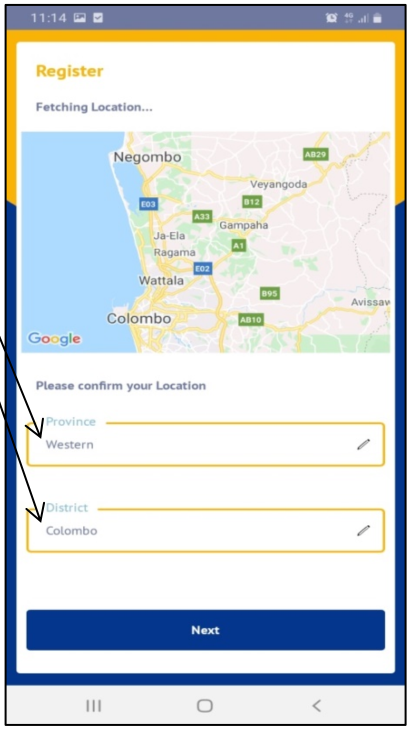

4. Enter the relevant information to the following screen and press '**Sign up**'

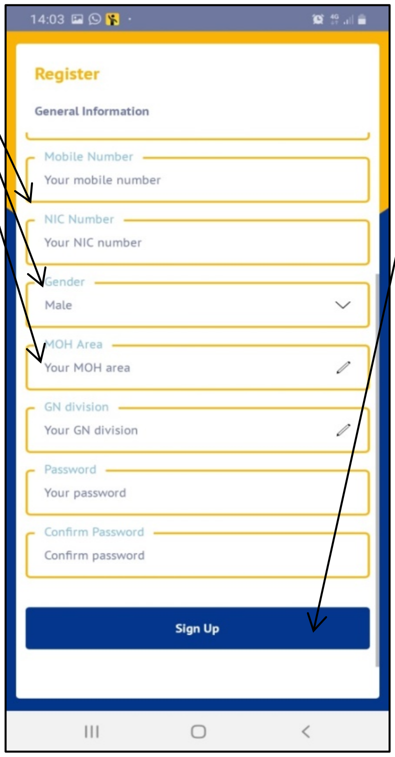

5. Await till you are approved by the System Administrator or District Health Education Officer. This may take up to 1 week.
6. Log in using your **NIC number** and **Password**

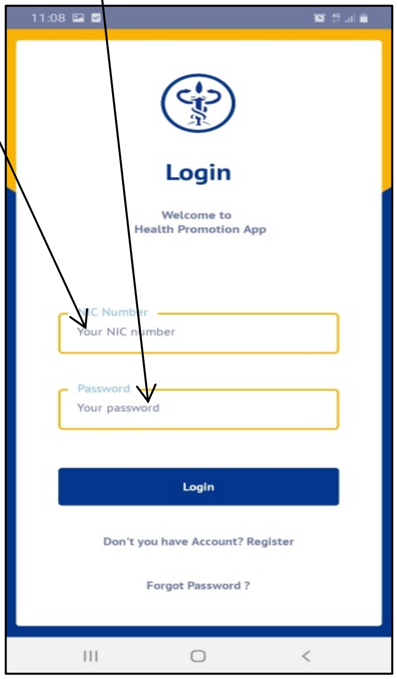

D: How to register a Mothers' Support Group

1. In the **Home screen/ Main menu** select '**Add Setting**'

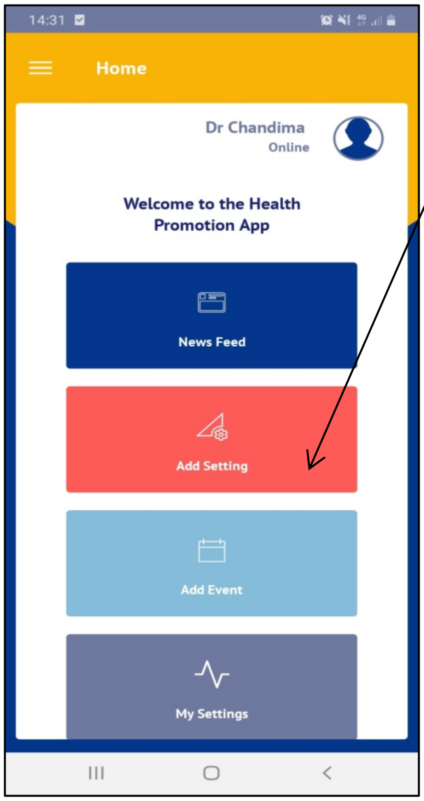

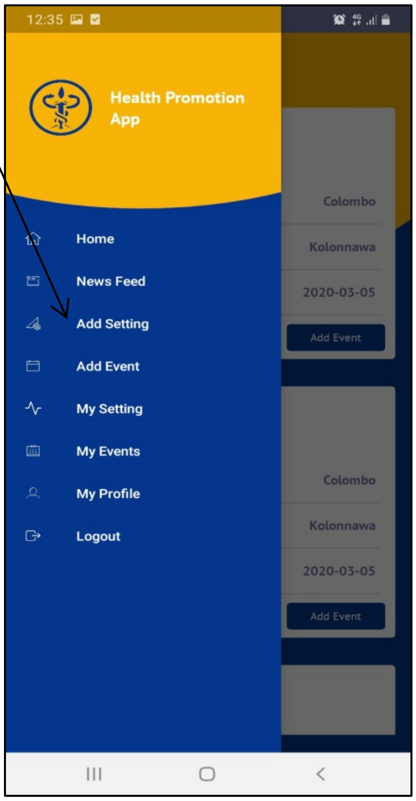

2. Select '**Mothers' Support Group**'.

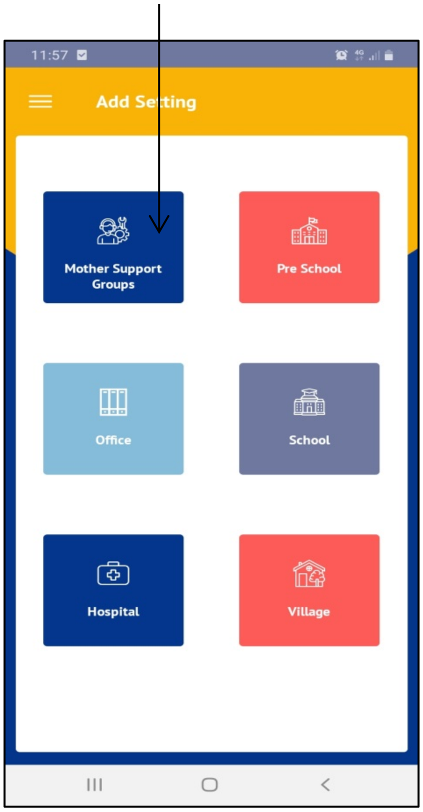

3. In the location confirmation screen select the **Province** and **District** and press **Next**

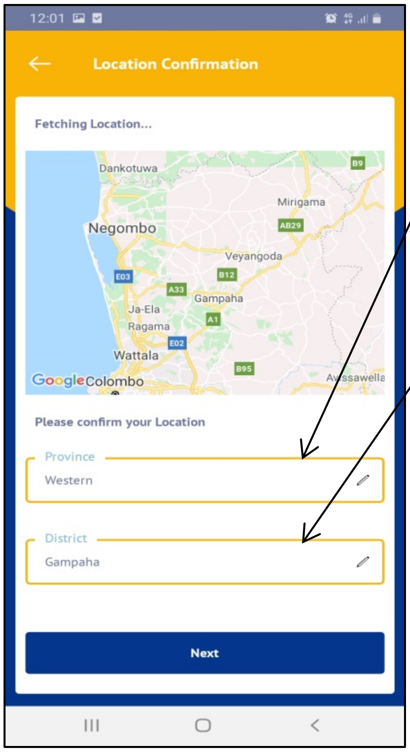

4. Fill in the relevant data and press '**Next**'

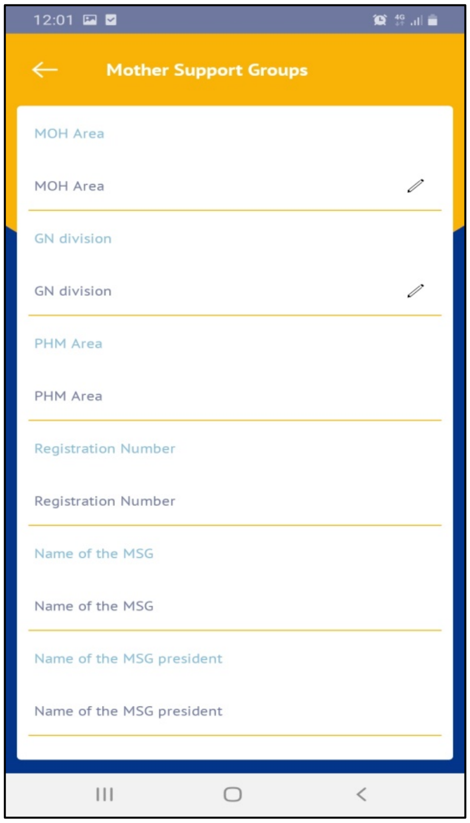

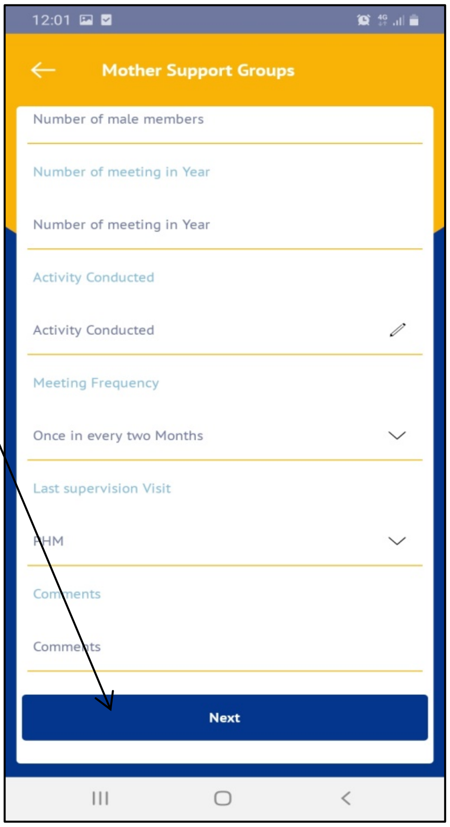

5. Select ‘**Upload image**’ to upload logo/picture selected from your gallery and press ‘**Next’** to complete.

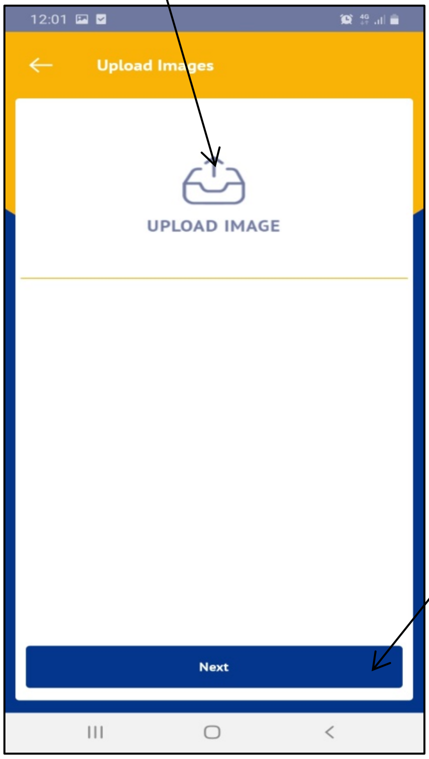

6. To confirm that you have successfully added the Mothers’ Support Group, go to **Home screen / Main menu** and select ‘**My Settings**’ and the setting will appear as in the following screen.

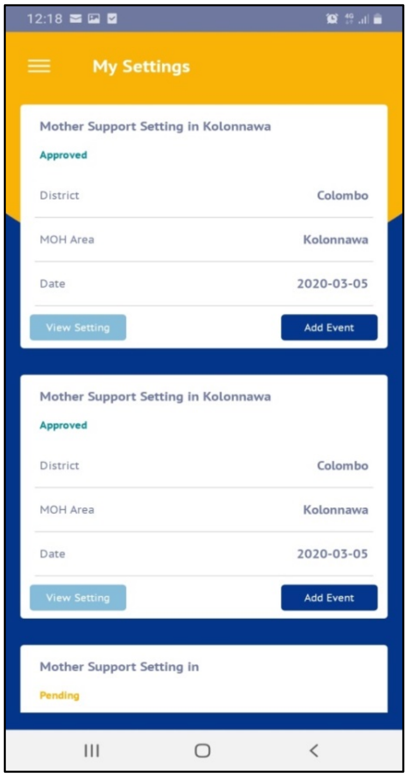

E: How to add an event conducted by the Mothers' Support Group

Select '**Add event**' in the **Home screen/ Main menu**

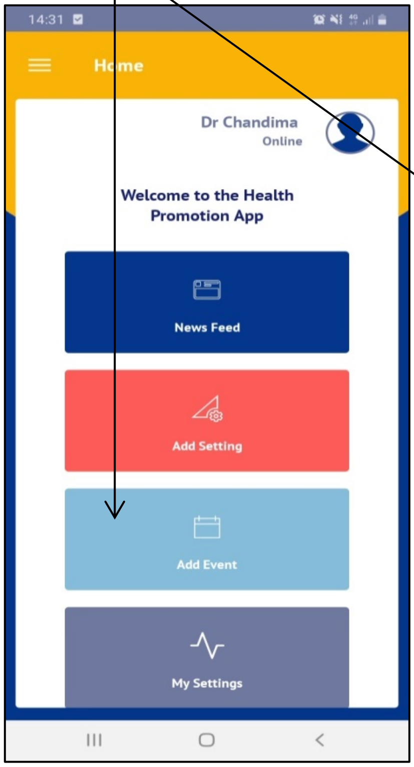

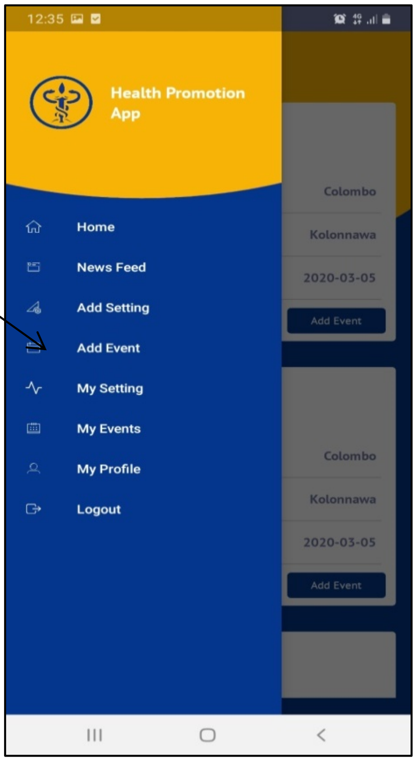

1. Fill in the relevant information and photos/ images if available and **submit**.

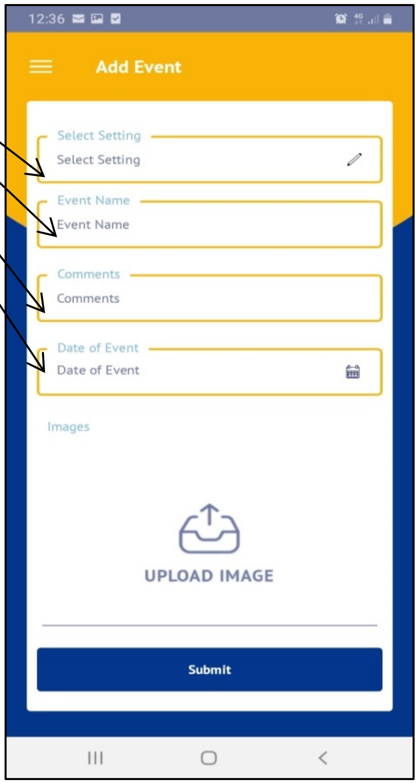

2. To confirm that you have successfully added the event, go to **Home screen/ Main menu** and select '**My Events**' and the event will appear as in the following screen.

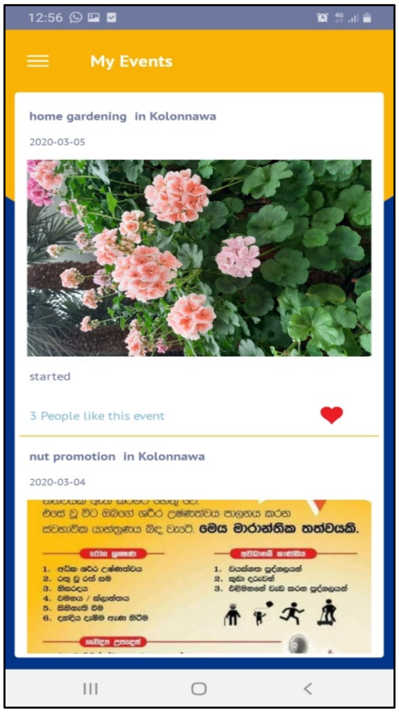

F: How to look at the latest events

1. Select ‘**News Feed** ’ in the **Home screen/ Main menu**

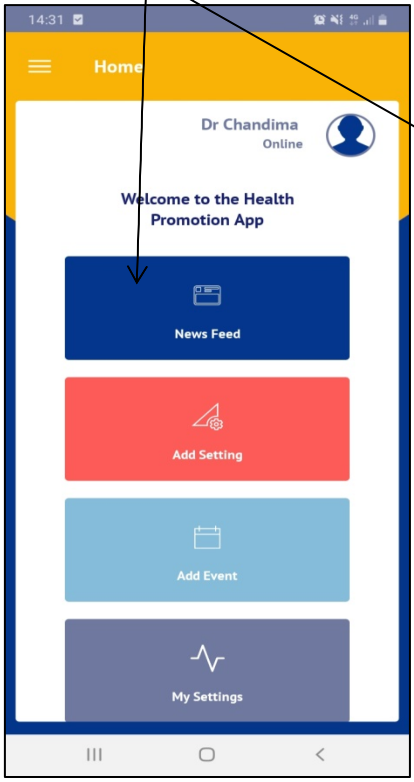

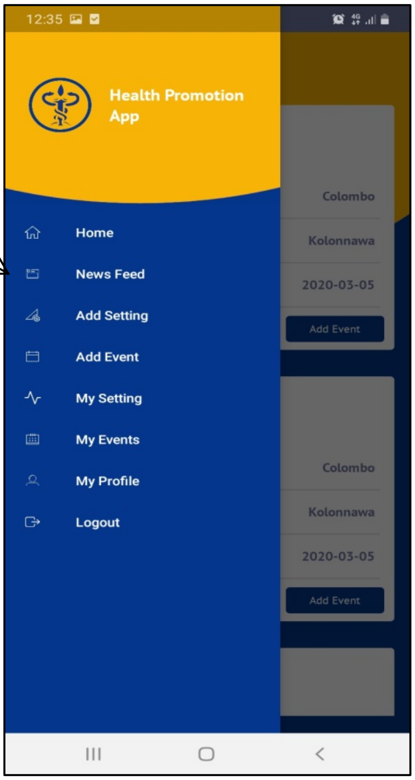

2. Most recent events will appear as follows

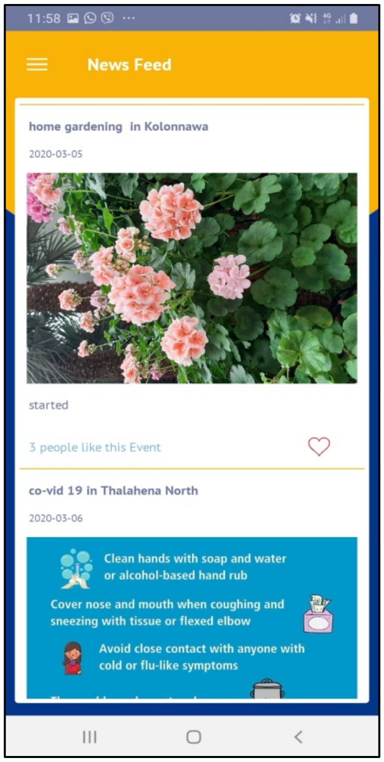

Family Health & Nutrition Communication Unit

Health Promotion Bureau
