# Supplementary material for: Revisiting health promotion settings: An innovative model from Sri Lanka to integrate healthy settings using mHealth
Source: Health Promot Perspect. 2022 May 29;12(1):28–33. doi: 10.34172/hpp.2022.04 (PMC9277291; doi:10.34172/hpp.2022.04)
Supplement: Supplementary file 2 — Supplementary files 1 and 2 contain training guides for the mobile app and the dashboard. [file hpp-12-28-s002.pdf]

INSTRUCTION MANUAL
HEALTH PROMOTION APP- DASHBOARD

Version 1.0

Family Health & Nutrition Communication Unit

Health Promotion Bureau

Table of Content

1 HOW TO LOGIN INTO THE DASHBOARD OF THE HEALTH PROMOTION APP? 2

1.1 LOGIN INTO AN EXISTING ACCOUNT USING PROVIDED PASSWORD 2
1.2 WHAT TO DO IF YOU HAVE FORGOTTEN THE PASSWORD PROVIDED? 4
1.3 HOW TO REGISTER FOR MOBILE APPLICATION USING THE WEB INTERFACE? 6
1.4 LOGGING OUT 7

2 DASHBOARD 9

2.1 SECTIONS PANEL 9
2.2 YOUR PROFILE 10
2.3 NOTIFICATIONS 10
2.4 SUMMARY PANEL 11
2.5 RECENT ACTIVITIES (EVENTS) 11
2.6 SETTING SUBMISSIONS (GEO LOCATIONS) 12
2.7 MONTHLY EVENT SUMMARY 13

3 MANAGE USERS 15

3.1 ENTERING TO THE SECTION 15
3.2 EDITING AN USER PROFILE 15
3.3 DELETING A USER PROFILE 16

4 MANAGE SETTINGS 17

4.1 ENTERING TO THE SECTION 17
4.2 SWITCH BETWEEN COMMUNITY ENGAGEMENT GROUP/ SETTINGS 17

5 MANAGE NEWS 20

5.1 ENTERING TO THE SECTION 20
5.2 USER INTERFACE 20
5.3 ADDING A NEW ‘NEWS’ THROUGH THE DASHBOARD 21

1 How to Login into the Dashboard of the Health Promotion App?

1.1 Login into an existing account using provided password

Step 1: Type, http://hpb.encyte.io in the address bar in your web browser. (We recommend Chrome browser for a better user experience).

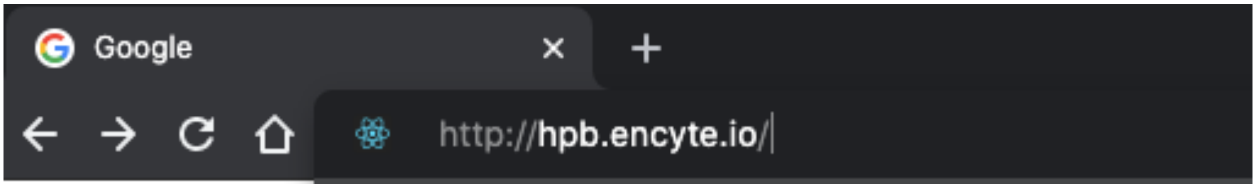

Then press "Enter" key in your keyboard. You will be directed to the login page.

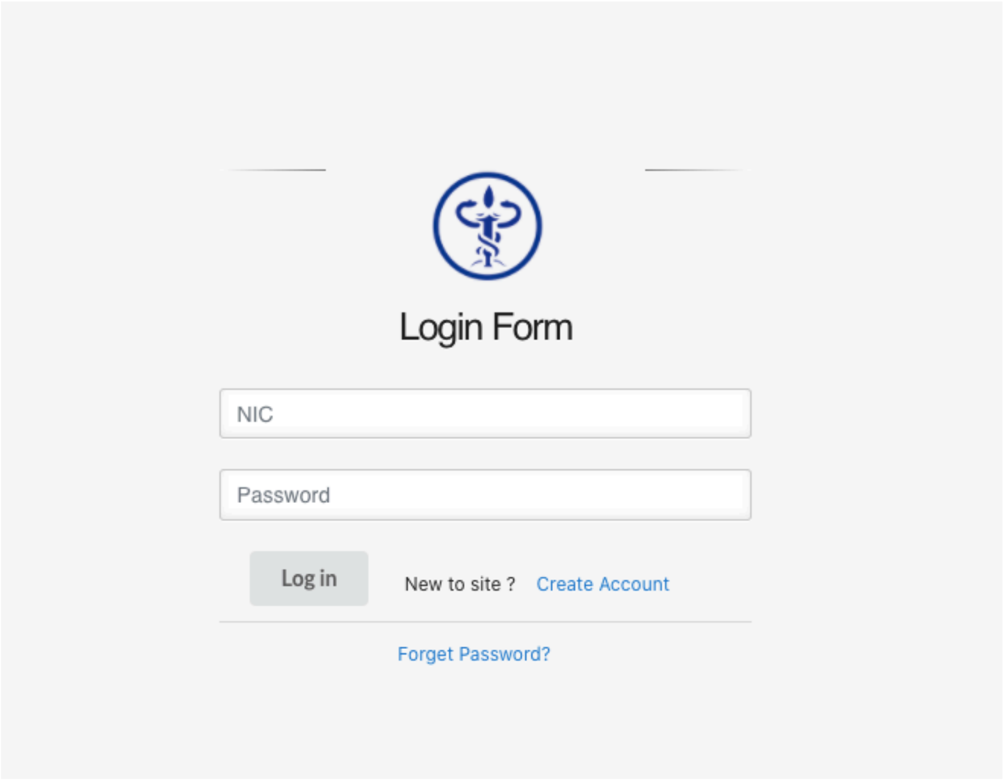

Step 2: Enter your National Identity Card Number (NIC) and the password provided by the system administrator (during pre-registration) here.

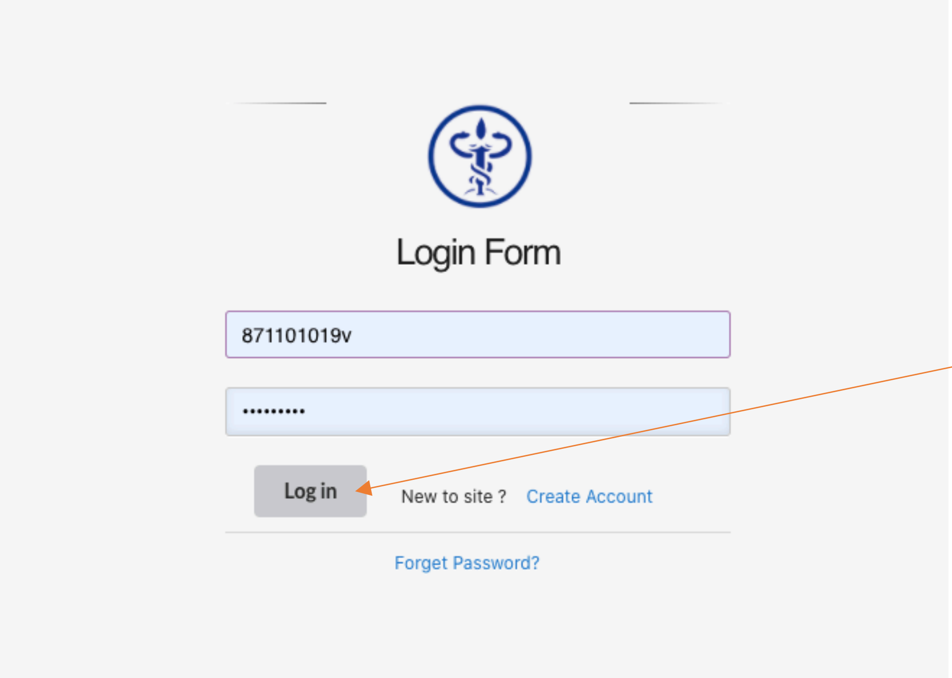

Press 'Enter" key in the keyboard or click here after inserting NIC and the password provided.

Then You will be directed to the Health Promotion App – Dashboard.

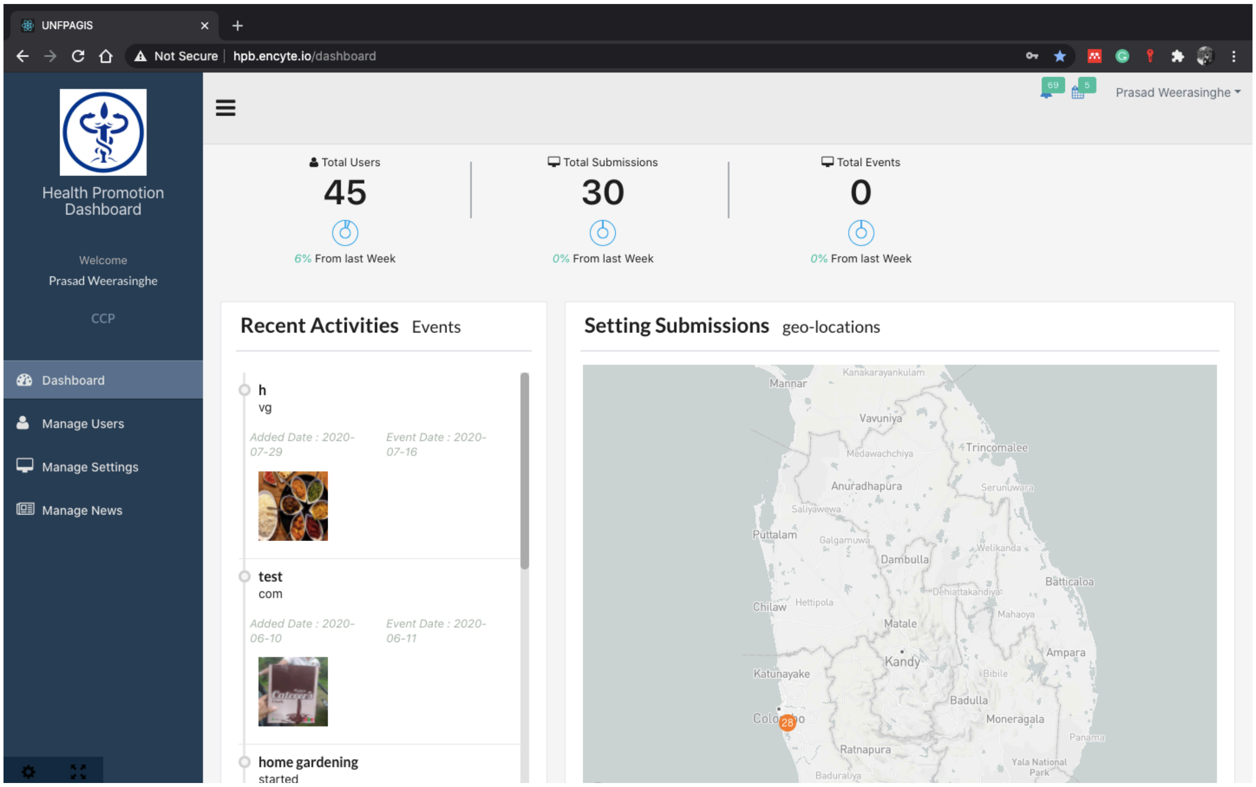

1.2 What to do if you have forgotten the password provided?

Step 1: Click on the "Forgot Password?" option in the login page.

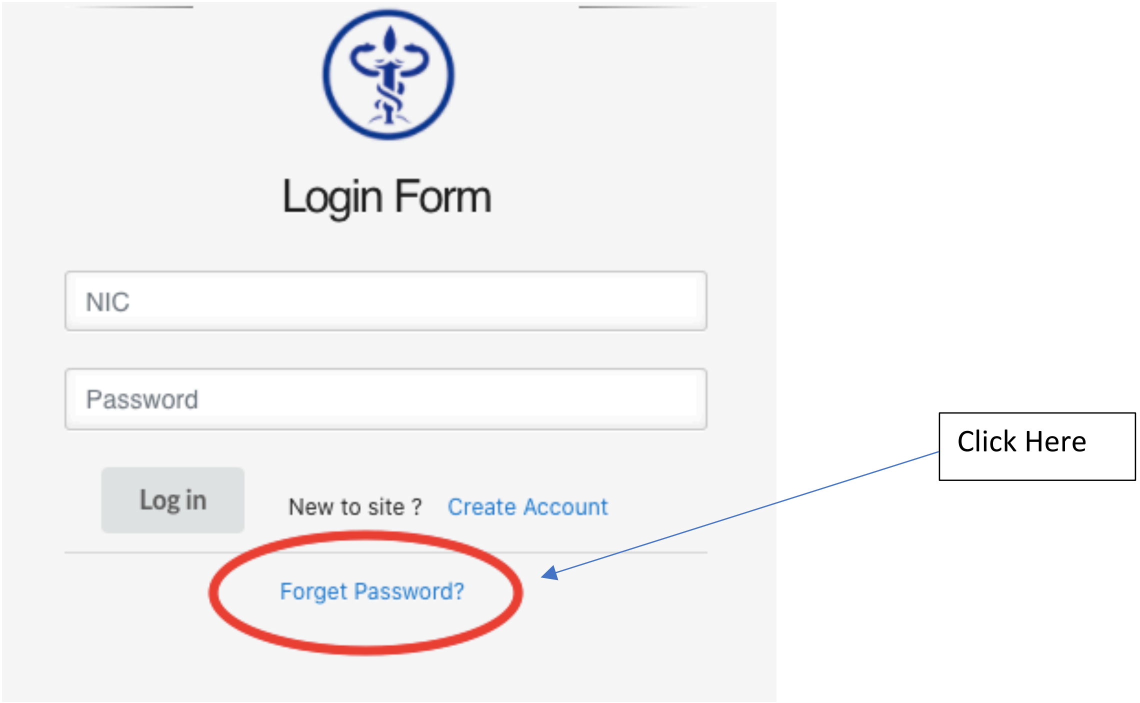

Step 2: Enter your NIC in the next dialog box and click on 'submit'.

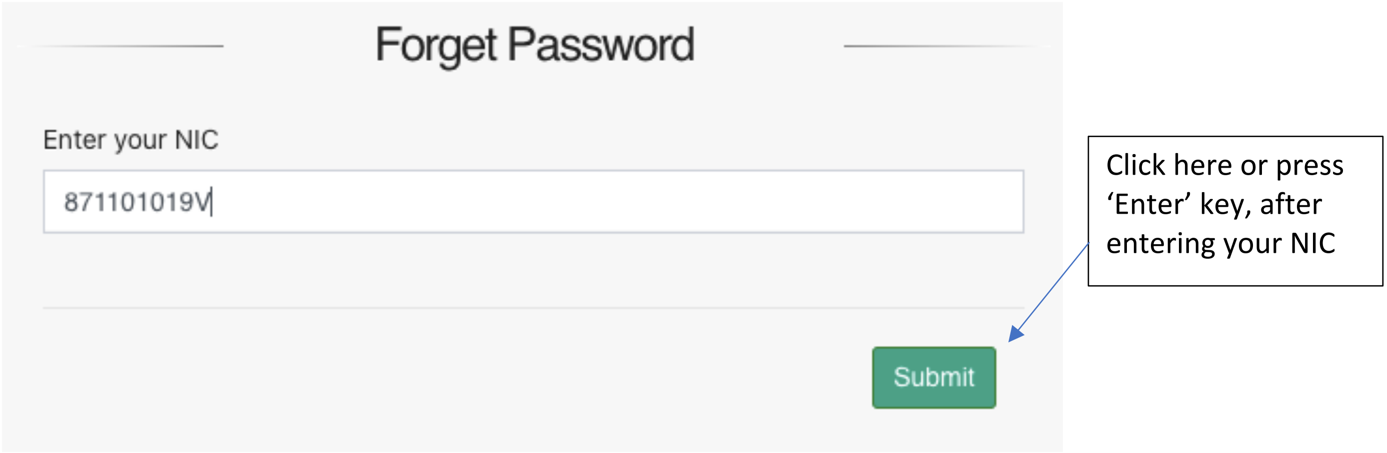

Step 3: Check your email (provided during pre-registration) to get the ‘passcode’.

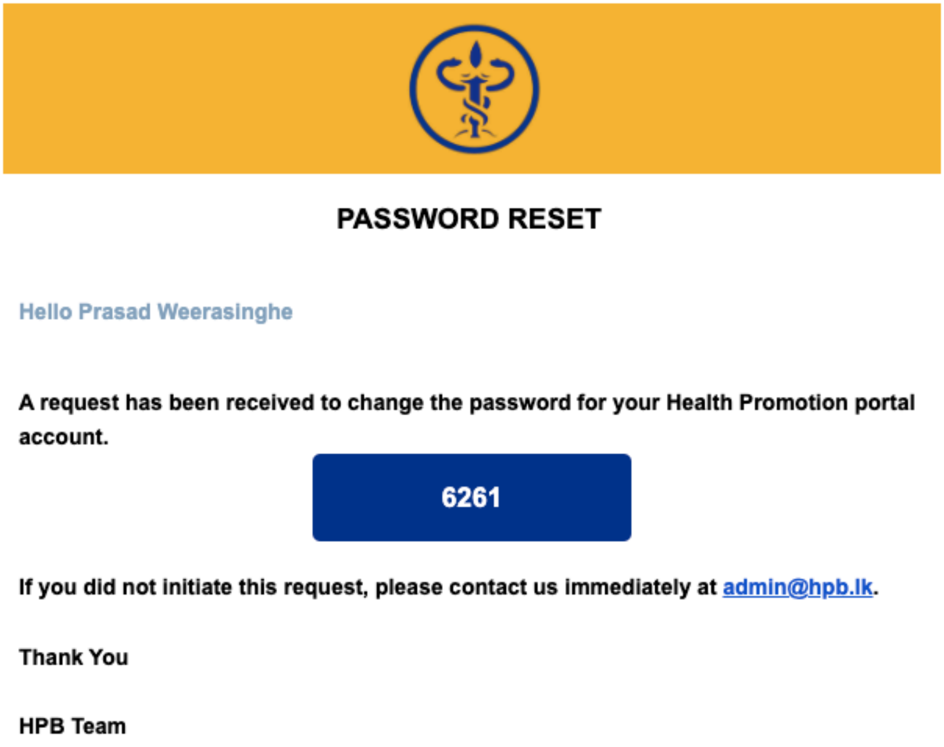
PASSWORD RESET

Hello Prasad Weerasinghe

A request has been received to change the password for your Health Promotion portal account.

6261

If you did not initiate this request, please contact us immediately at admin@hpb.lk.

Thank You

HPB Team

Step 4: Enter received Passcode in the dialog box and submit.

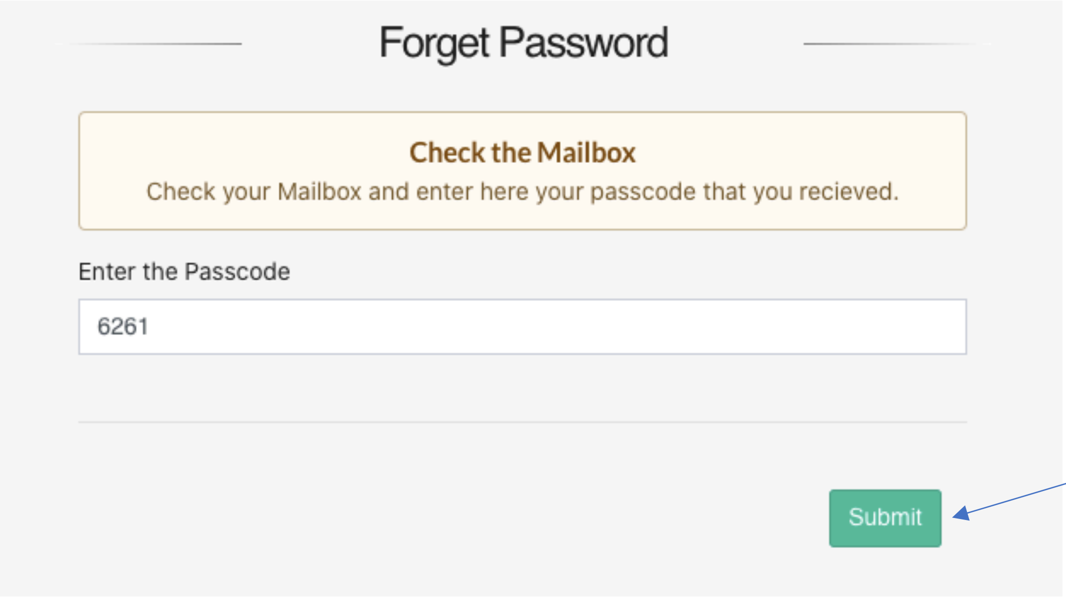

Click here or press ‘Enter’ key, after entering your passcode

Step 5: Enter your new password and Submit.

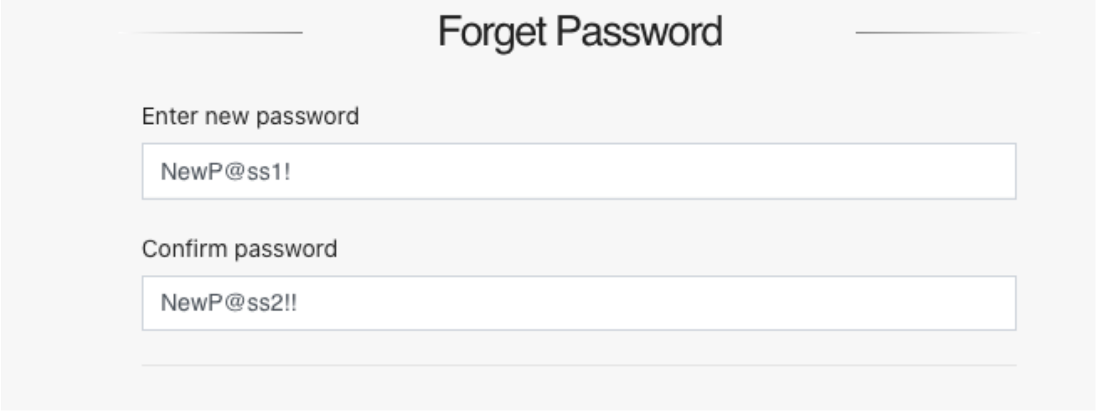

Step 6: Enter your new password and log in as usual.

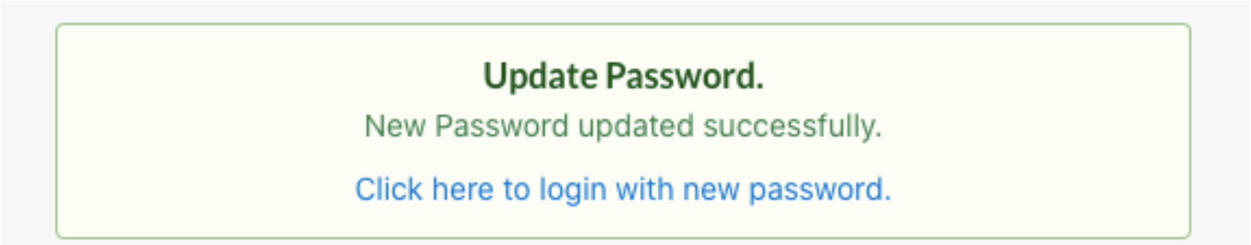

1.3 How to register for mobile application using the web interface?

Step 1: Click on the 'Create Account' option in the logging screen.

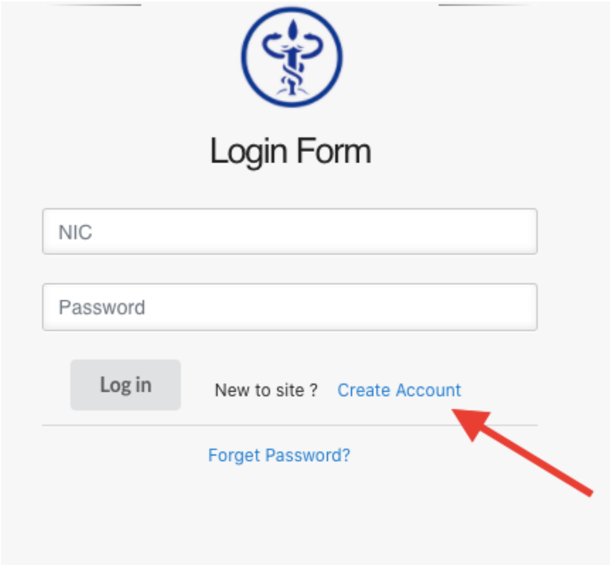

Step 2: Fill the form and submit to get registered. Log in to the mobile app using this account.

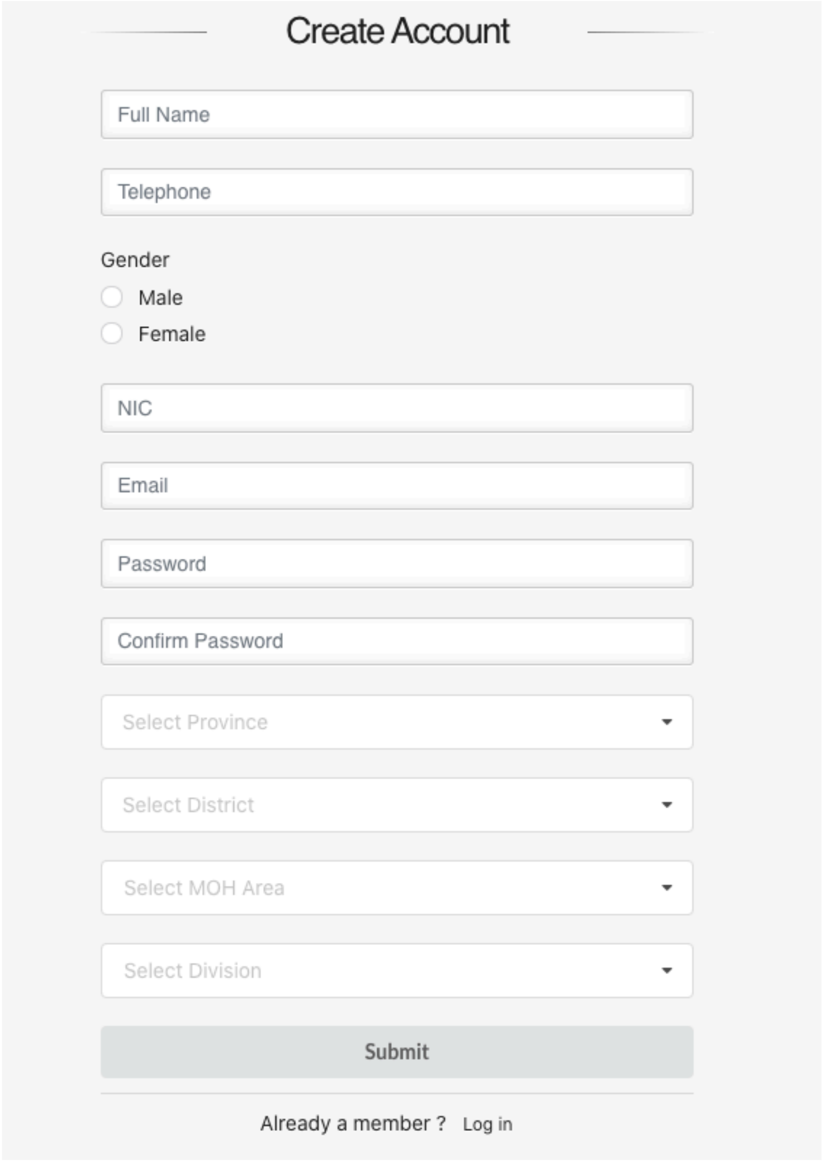

** This is not for registration of the higher-level users who have access to the Health Promotion App Dashboard.
***All the Higher-level user accounts need to be created by a System Admin.

1.4 Logging out

Click on your 'Profile Name' on the right upper corner.

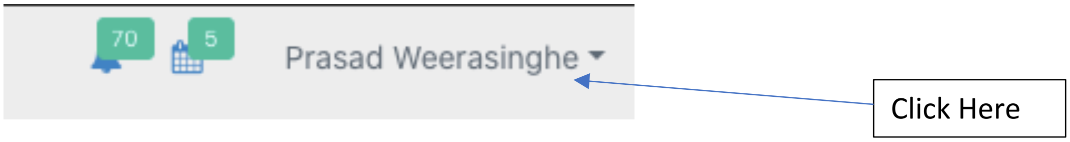

Then click on the 'logout' option.

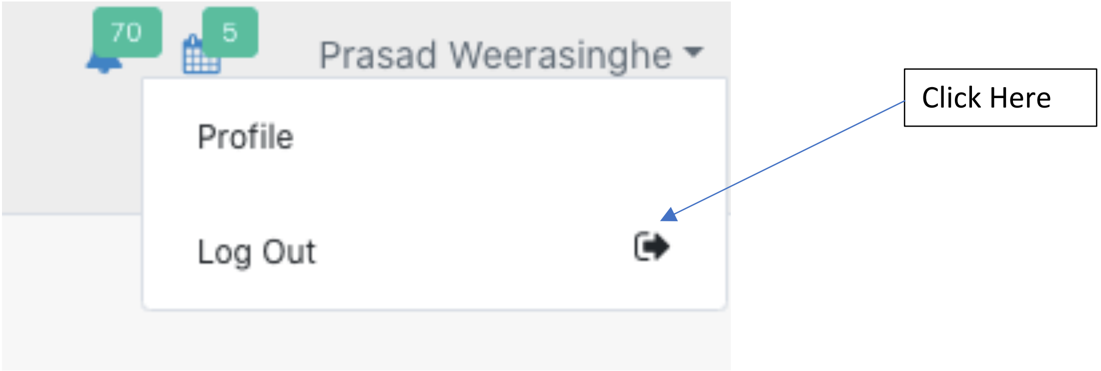

2 Dashboard

2.1 Sections Panel

After login you will be in the 'Dashboard' tab by default. To see other sections of the web interface click on the following icon (left upper corner).

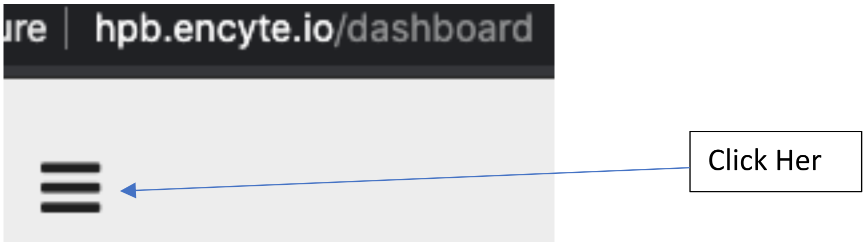

Then you will see the hidden 'sections panel' with highlighted 'dashboard' section.

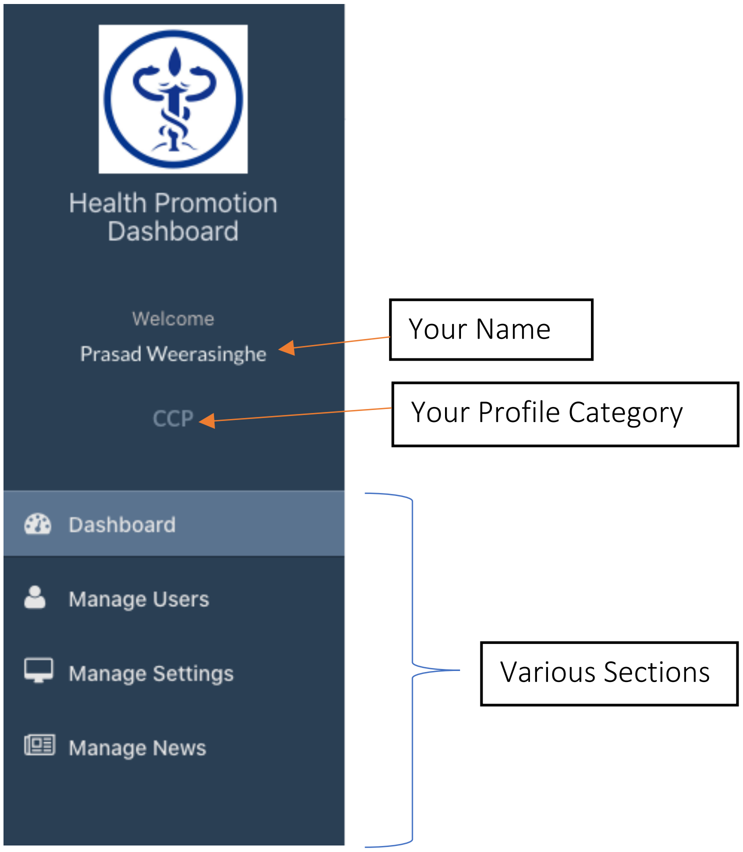

Let's discuss each and every area of this 'Dashboard' section now.

2.2 Your Profile

You can edit some of your profile details. First click on your profile name on right upper corner of the interface.

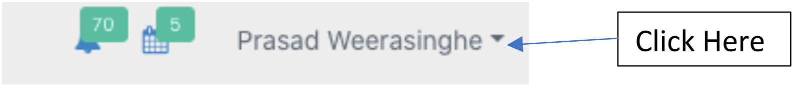

Then Click on the 'profile' option in the drop-down menu.

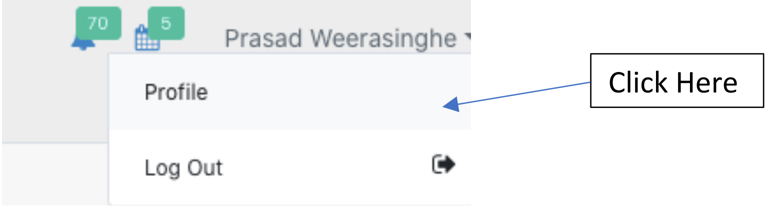

Now you can see your user profile. Only some of the details are editable .

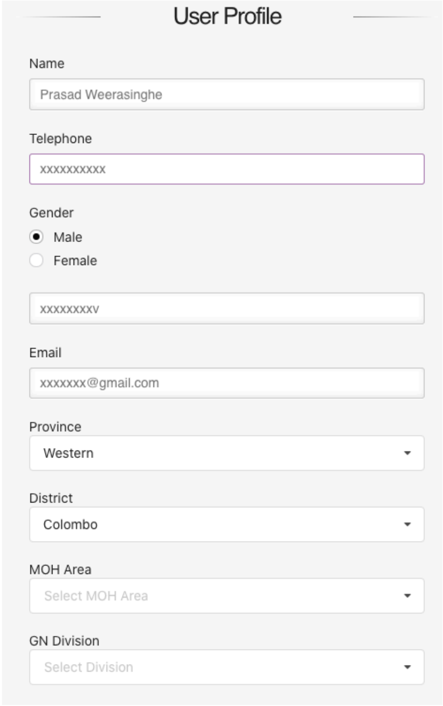

There are various profile categories.

2.3 Notifications

There are two notification icons on the right upper corner. (Left – Bell, Right – Calander)

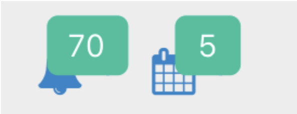

Bell icon will provides notifications about newly registered users. Click on the bell icon and see the recently registered users list.

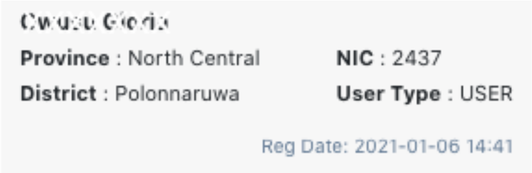

Calendar Icon will give notifications about newly registered 'Health Promotion Settings' (Will be discussed in detail later). Click on the calendar Icon and see the recently registered settings list.

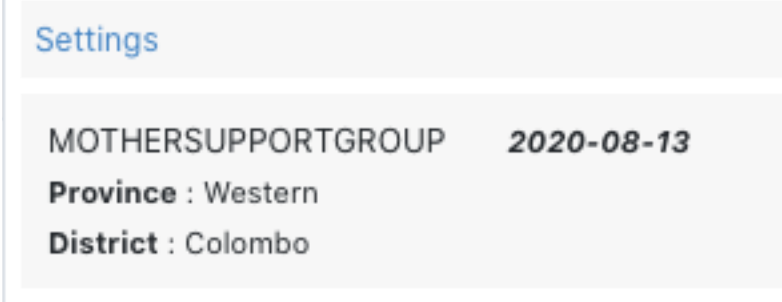

2.4 Summary panel

This will provide a summary about the area assigned for you. From left, Total users, Total Submissions (Settings) and Total events. Under each there will be percentage from last week.

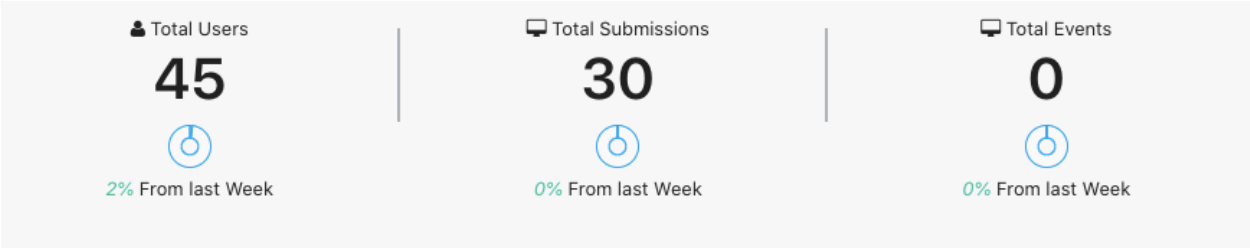

2.5 Recent Activities (Events)

In this section you can see the recent activities/events posted by various settings.

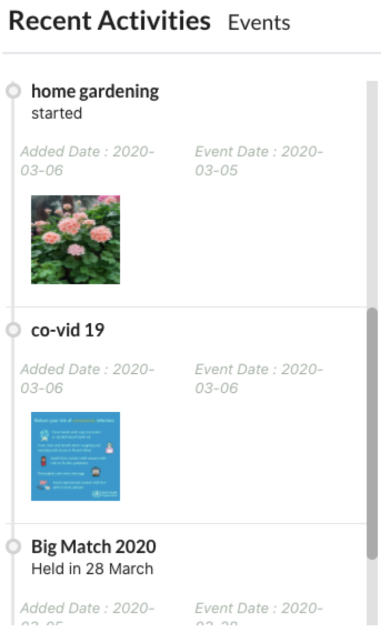

You can scroll and see more recent events

2.6 Setting submissions (Geo locations)

In this map box you can see geological locations of the submitted settings under your purview. It’s possible to zoom in or out using scroll key of the mouse or touch pad gesture.

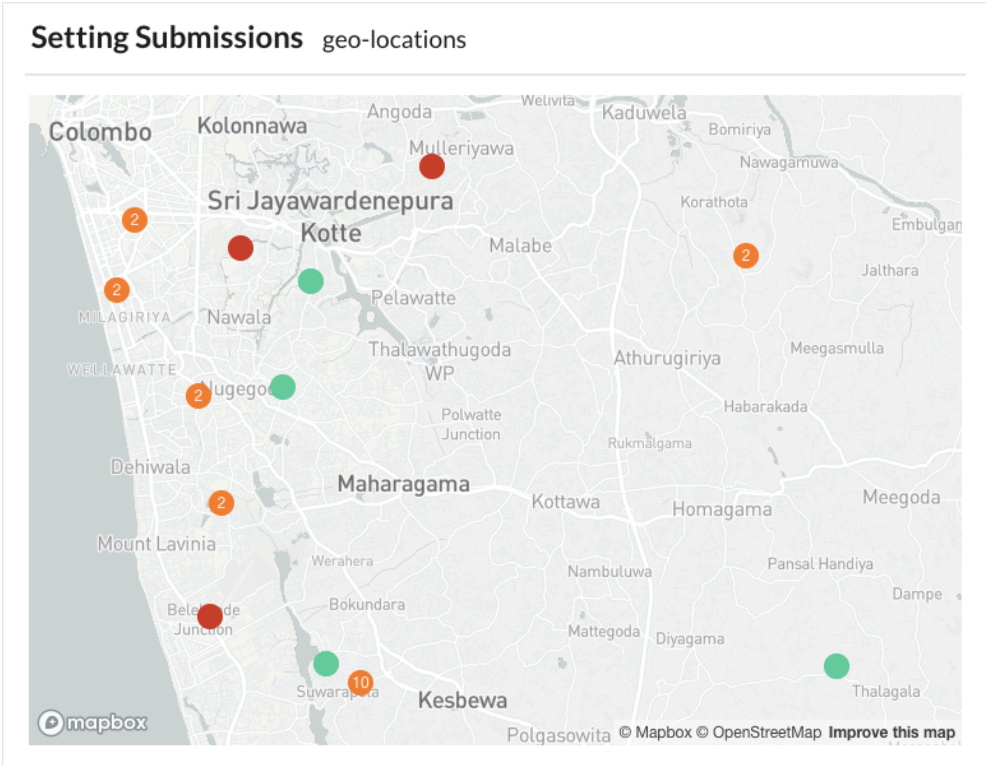

2.7 Monthly event summary

In this graph you can see the total number of events by month.

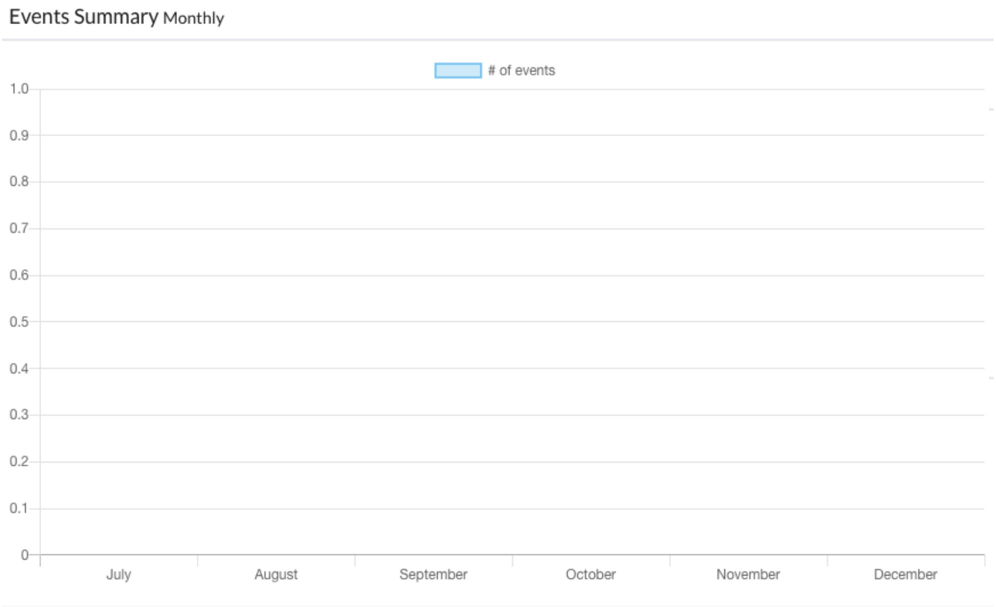

There are five health promotion settings. Namely Mother Support Group, Pre-Schools, Schools, Work Places and Villages.

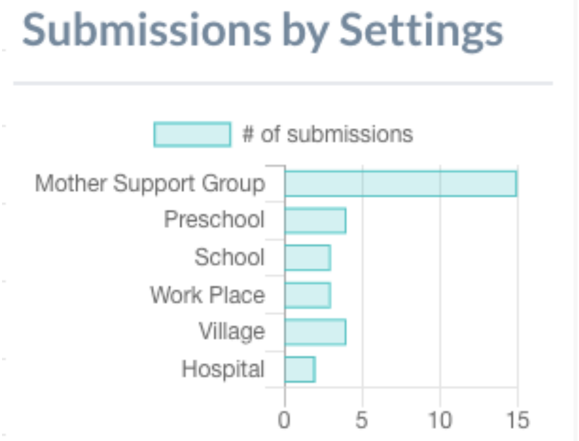

Number of submissions from each health promotion setting is summarized in this graph.

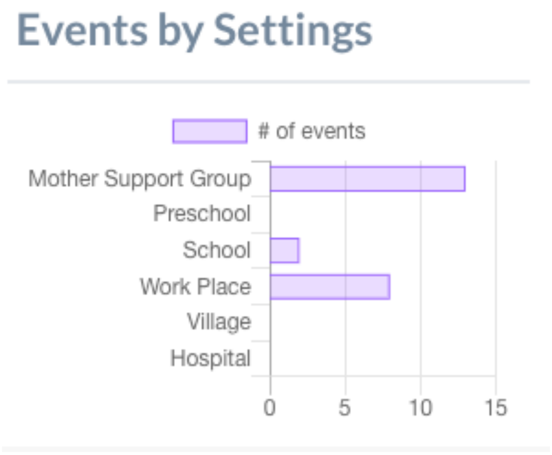

Number of events posted by each setting is summarized in this graph

District level summary of activities posted by each setting is displayed in this graph.

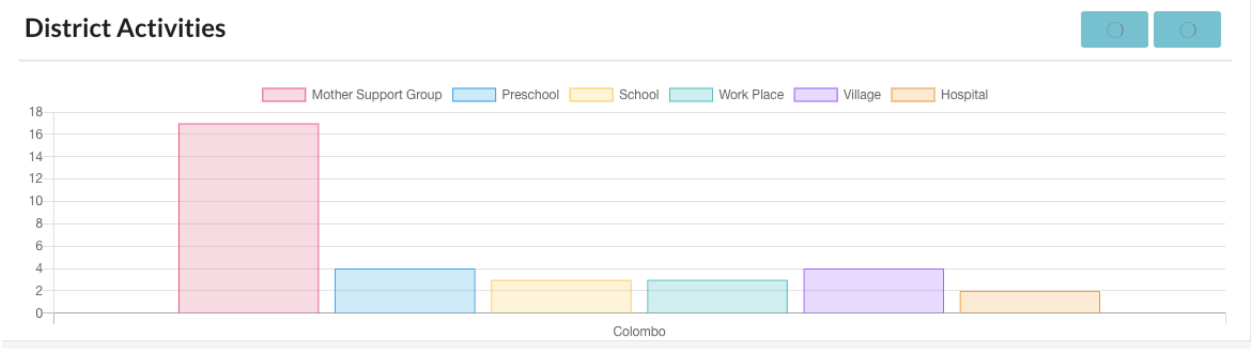

3 Manage Users

3.1 Entering to the Section

You can enter this section by clicking the ‘manage users tab in the left side selection menu.

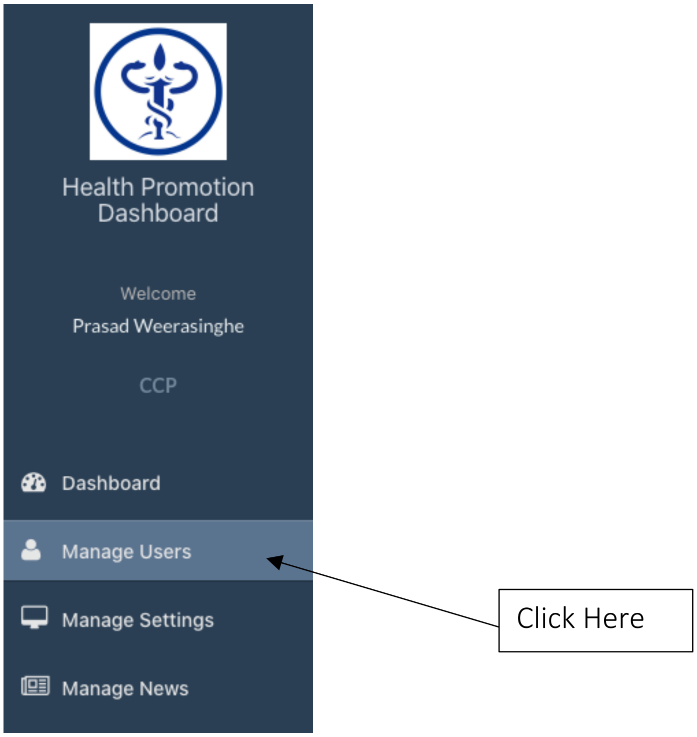

Then you will be able to see the list of users registered from your area.

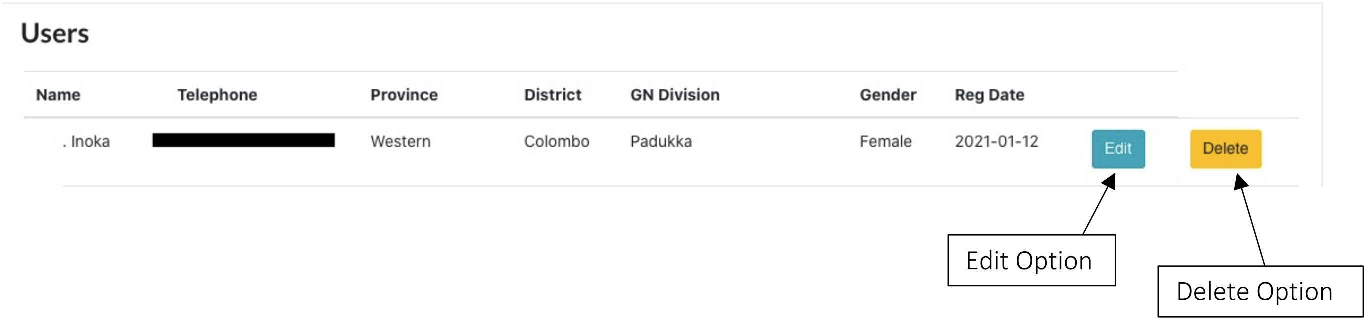

3.2 Editing an user profile

By clicking on the Edit option you can enter to the relevant user profile and edit certain details.

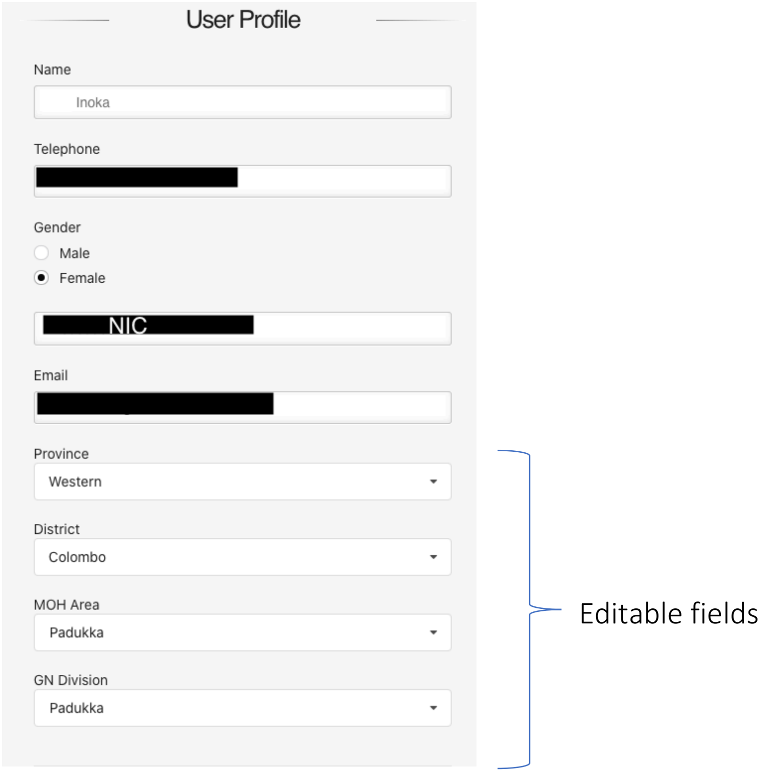

Edit relevant fields carefully. This has to be done only when the current details in the profile are incorrect.

3.3 Deleting a user profile

You can delete a user profile by clicking the ‘Delete’ option. Please be careful when you delete a user profile to select the correct one. This action is irreversible.

4 Manage Settings

4.1 Entering to the section

You can enter to the ‘Manage settings’ section by clicking that option in the left side selection menu.

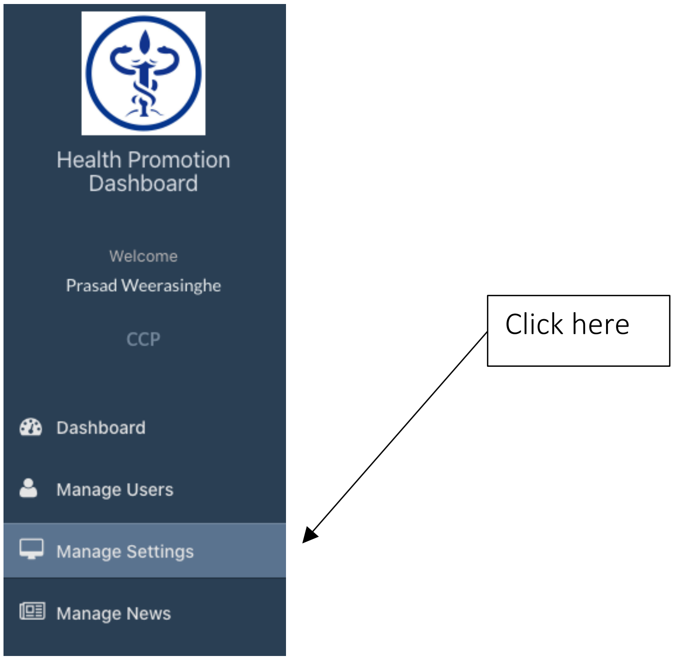

4.2 Switch between Community Engagement group/ Settings

You can switch between settings by clicking the relevant tab at the top of the section.

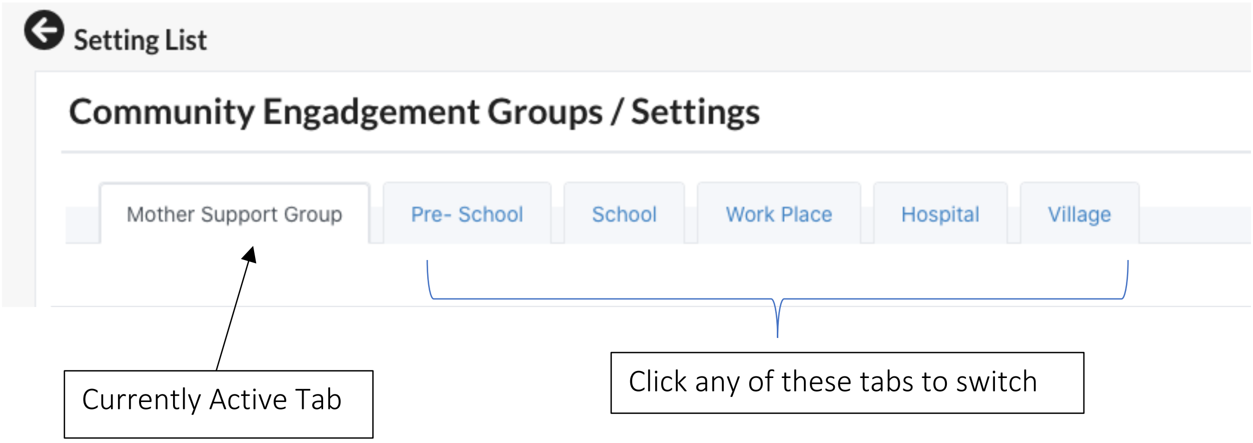

Layout of each settings tab is similar.

4.3 Options available for edit 'Settings'

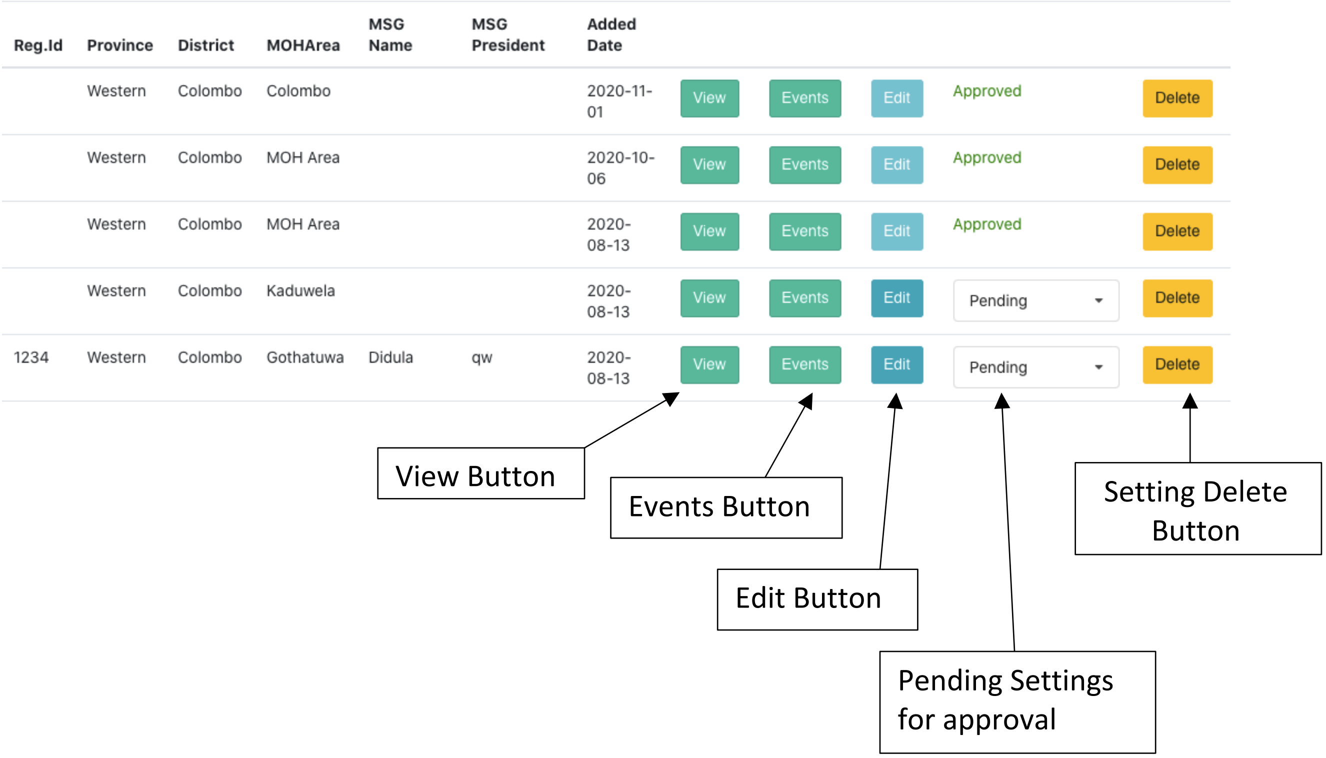

Reg.Id	Province	District	MOHArea	MSG Name	MSG President	Added Date					
	Western	Colombo	Colombo			2020-11-01	View	Events	Edit	Approved	Delete
	Western	Colombo	MOH Area			2020-10-06	View	Events	Edit	Approved	Delete
	Western	Colombo	MOH Area			2020-08-13	View	Events	Edit	Approved	Delete
	Western	Colombo	Kaduwela			2020-08-13	View	Events	Edit	Pending	Delete
1234	Western	Colombo	Gothatuwa	Didula	qw	2020-08-13	View	Events	Edit	Pending	Delete

View – By clicking this you can view the Setting profile

Events – This will show you the events posted from that setting

Edit – Can edit profiles of settings pending for approval using this button.

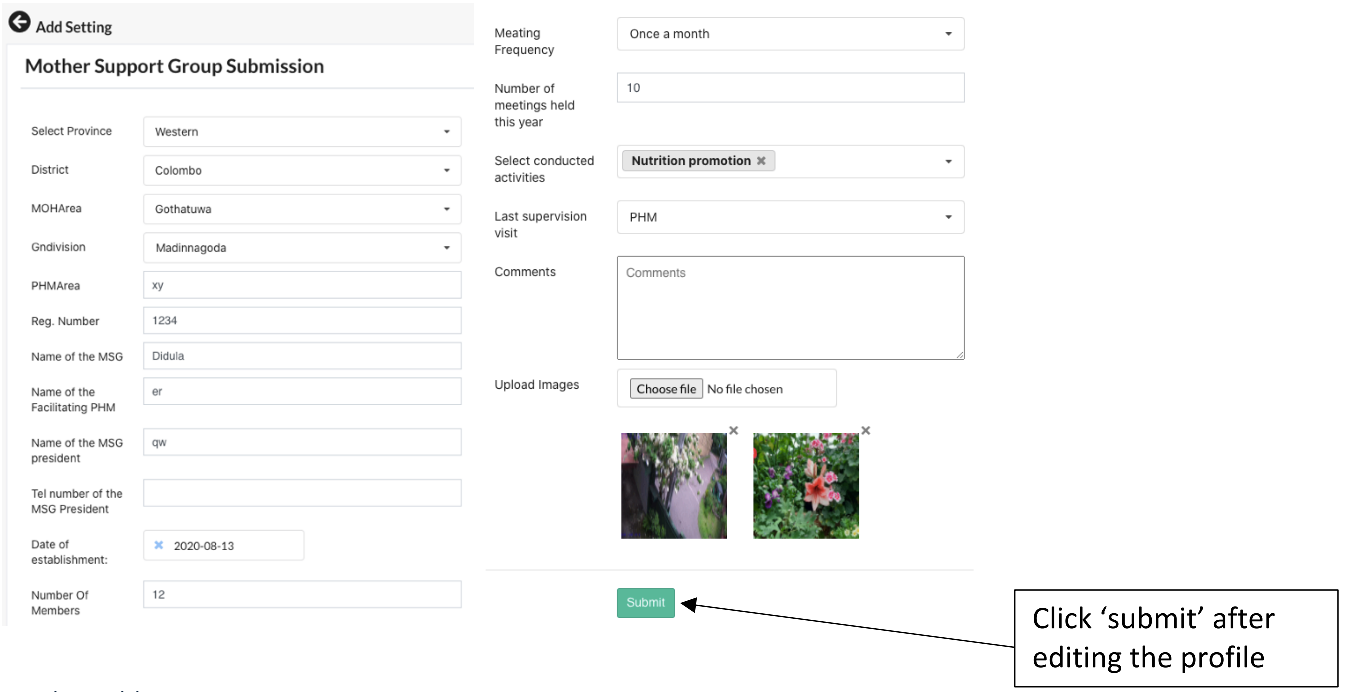

Pending settings for approval – Click on the 'Pending' option to accept or reject the setting submission.

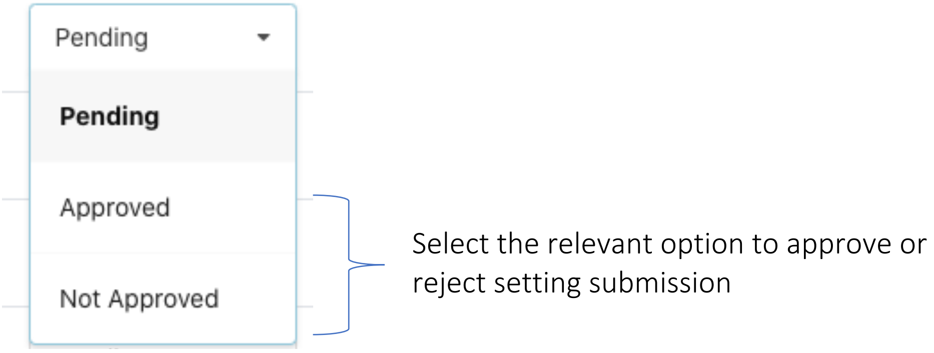

Delete - By clicking this you can delete a registered or a pending 'setting'.

5 Manage News

5.1 Entering to the section

You can access the 'Manage News' section by clicking the title at the left sided selection menu.

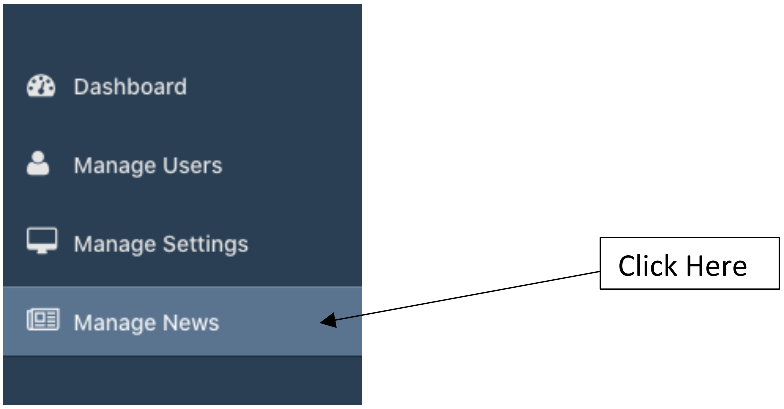

Then you can see the user interface.

5.2 User interface

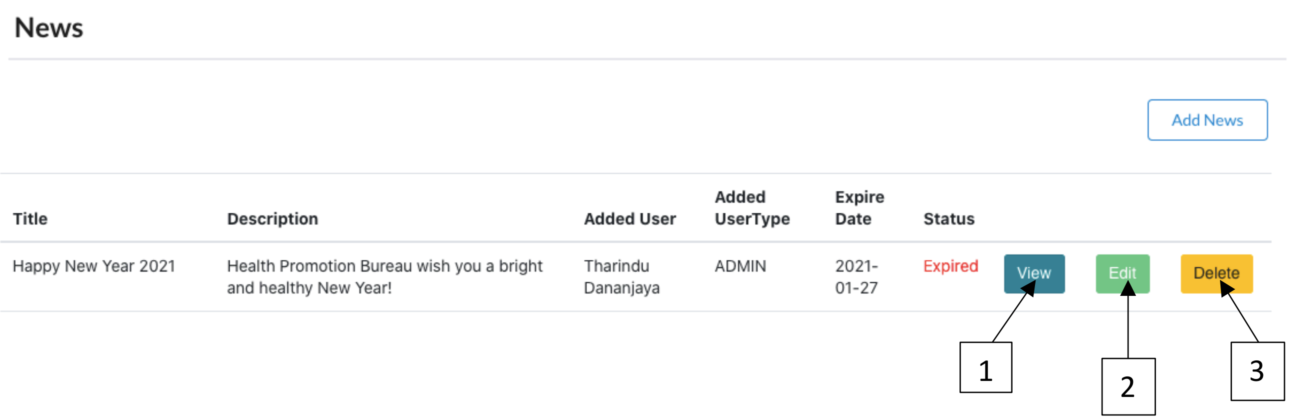

For each news,

Title, Description, Added user and type, expiry date and the current status is displayed in the menu.

There are three options available to manage a news.

1. View – view the news

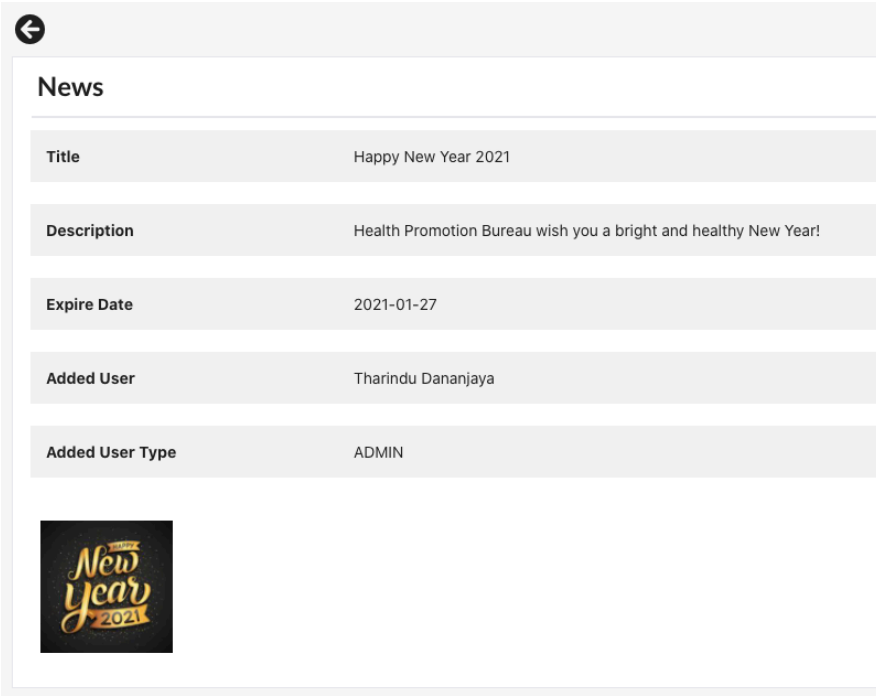

2. Edit - edit the already posted news
3. Delete – Delete the news

5.3 Adding a new 'news' through the dashboard

Step 1 – Click on the Add News Button on the right upper corner in the user interface

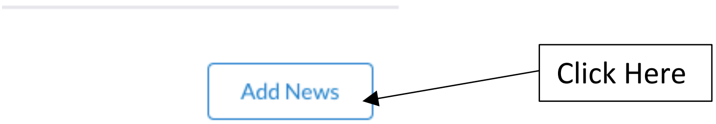

Step 2 – Fill the Title and the content of the news.

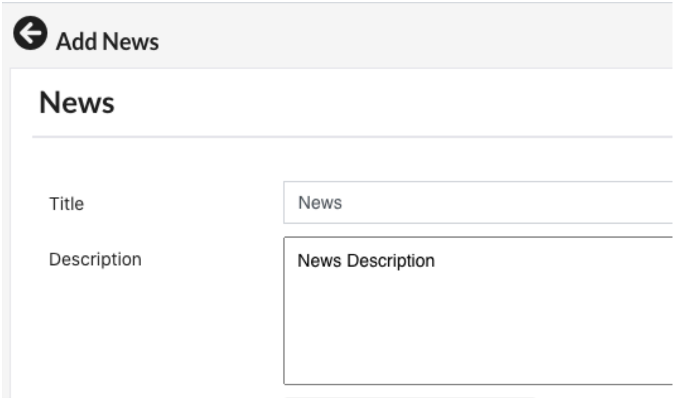

Step 3 – Set an expiry date for the news if you need (optional).

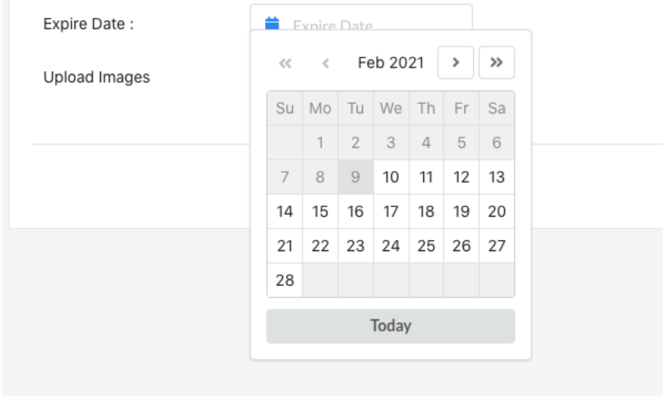

Step 4 – Upload Image related to the news (optional)

Choose the image file through the ‘choose file’ button. Then click in ‘Click here to Upload”

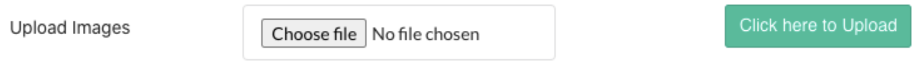

Step 5 – Submit the news by clicking the ‘submit’ button.

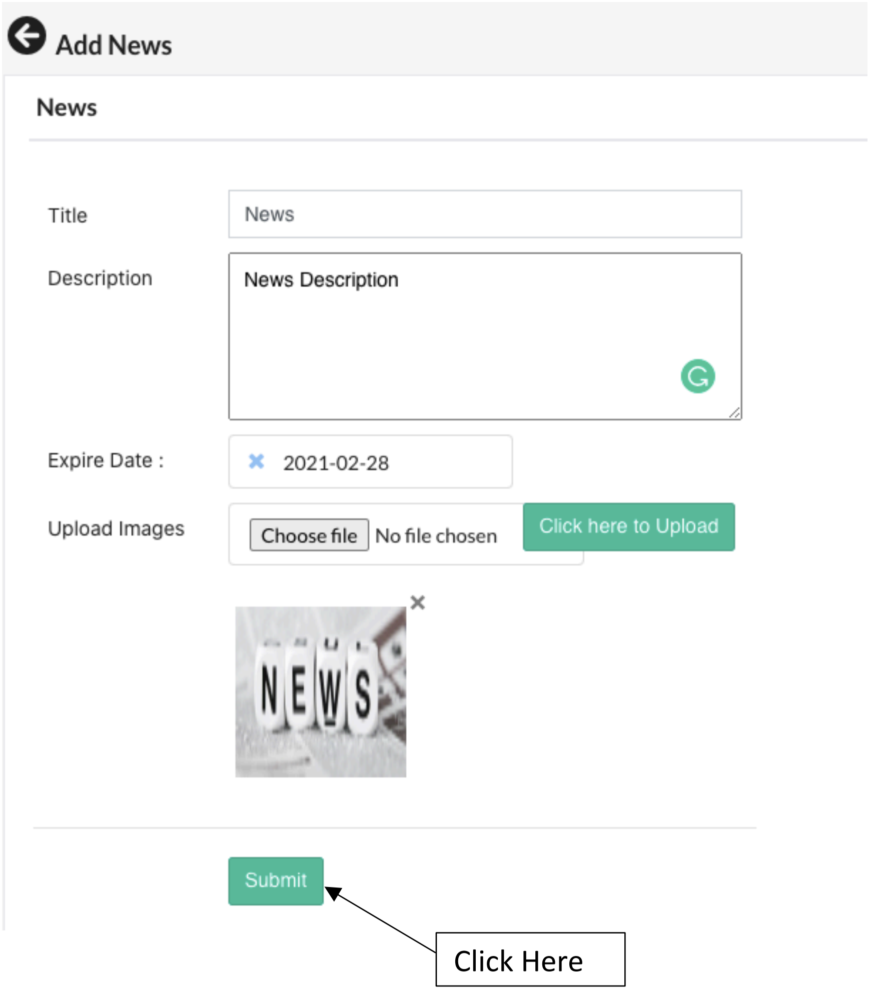
Add News
News
Title
News
Description
News Description
Expire Date :
2021-02-28
Upload Images
Choose file
No file chosen
Click here to Upload
NEWS
Submit
Click Here
